# Adaptations to Climate-Mediated Selective Pressures in Humans

**DOI:** 10.1371/journal.pgen.1001375

**Published:** 2011-04-21

**Authors:** Angela M. Hancock, David B. Witonsky, Gorka Alkorta-Aranburu, Cynthia M. Beall, Amha Gebremedhin, Rem Sukernik, Gerd Utermann, Jonathan K. Pritchard, Graham Coop, Anna Di Rienzo

**Affiliations:** 1 Department of Human Genetics, University of Chicago, Chicago, Illinois, United States of America; 2 Department of Anthropology, Case Western Research University, Cleveland, Ohio, United States of America; 3 Department of Internal Medicine, Addis Ababa University, Addis Ababa, Ethiopia; 4 Laboratory of Human Molecular Genetics, Department of Molecular and Cellular Biology, Institute of Chemical Biology and Fundamental Medicine, Russian Academy of Sciences, Novosibirsk, Russia; 5 Institute for Medical Biology and Human Genetics, Medical University of Innsbruck, Innsbruck, Austria; 6 Howard Hughes Medical Institute, Chevy Chase, Maryland, United States of America; 7 Department of Evolution and Ecology and Center for Population Biology, University of California Davis, Davis, California, United States of America; University of Arizona, United States of America

## Abstract

Humans inhabit a remarkably diverse range of environments, and adaptation through natural selection has likely played a central role in the capacity to survive and thrive in extreme climates. Unlike numerous studies that used only population genetic data to search for evidence of selection, here we scan the human genome for selection signals by identifying the SNPs with the strongest correlations between allele frequencies and climate across 61 worldwide populations. We find a striking enrichment of genic and nonsynonymous SNPs relative to non-genic SNPs among those that are strongly correlated with these climate variables. Among the most extreme signals, several overlap with those from GWAS, including SNPs associated with pigmentation and autoimmune diseases. Further, we find an enrichment of strong signals in gene sets related to UV radiation, infection and immunity, and cancer. Our results imply that adaptations to climate shaped the spatial distribution of variation in humans.

## Author Summary

Classical studies that examined the global distributions of human physiological traits such as pigmentation, basal metabolic rate, and body shape and size suggested that natural selection related to climate has been important during recent human evolutionary history. We scanned the human genome using data for about 650,000 variants in 61 worldwide populations to look for correlations between allele frequencies and 9 climate variables and found evidence for adaptations to climate at the genome-wide level. In addition, we detected compelling signals for individual SNPs involved in pigmentation and immune response, as well as for pathways related to UV radiation, infection and immunity, and cancer. A particularly appealing aspect of this approach is that we identify a set of candidate advantageous SNPs associated with specific biological hypotheses, which will be useful for follow-up testing. We developed an online resource to browse the results of our data analyses, allowing researchers to quickly assess evidence for selection in a particular genomic region and to compare it across several studies.

## Introduction

Climatic factors like temperature and humidity play an important role in determining species distributions and they likely influence phenotypic variation of populations over geographic space. Several eco-physiological “rules” have been proposed to predict variation in body size, pigmentation and body dimensions as functions of climate or geography [1]–[3]. Many subsequent studies showed support for Bergmann's and Allen's rules both within (e.g. [4]–[7] and among species (e.g., [8]–[11]. Additional evidence for observed gradients in other phenotypes over space as well as observed correlations between phenotypes and ecological factors led Julian Huxley to define the term “cline” to refer to “a gradation in measurable characters” [12]. Huxley stressed the importance of distinguishing between phenotypic variation with a genetic basis and variation resulting simply from phenotypic plasticity.

Since human populations occupy a wide variety of environments with respect to climate, selective pressures are expected to vary greatly across geographic regions. Adaptations to spatially varying selective pressures are evident in the geographic distributions of many traits. For example, significant correlations exist between body mass and temperature [13]–[14], consistent with Bergmann's and Allen's Rules. Furthermore, there is evidence that human metabolism has been shaped by adaptations to cold stress from studies of arctic populations, which exhibit elevated basal metabolic rates compared to non-indigenous populations [15]. Like body mass, variation in skin pigmentation is strongly correlated with climate and geography, i.e. distance from the equator and solar radiation [16]–[17]. Lighter pigmentation is likely to be adaptive in high latitudes, in part, because UV light is needed to penetrate the skin to produce vitamin D [16]–[19], which is necessary for calcium absorption and bone growth.

For these ecoclines to be evolutionarily relevant, they must have a genetic basis. Several studies have examined the distributions of genetic variants in candidate genes for traits that vary with climate. Latitudinal clines of allele frequencies have been observed for several protein polymorphisms in humans (e.g. [20]–[21]). Furthermore, candidate gene approaches in humans as well as several other species support roles for selection at genetic variants that underlie phenotypic variation. For example, in humans, candidate gene studies have yielded evidence that variants involved in sodium homeostasis and energy metabolism are correlated with latitude and climate [22]–[24]. In addition to individual candidate genes, strong correlations between allele frequency and climate variables were found at high-density tagging SNPs in a set of 82 genes within the network associated with common metabolic disorders; in this study, the enrichment was assessed relative to a limited set of control SNPs [25]. In *Drosophila melanogaster,* variants involved in circadian rhythms, aging and energy metabolism are correlated with climate (e.g. [26]–[30]), in *Arabidopsis thaliana*, variants associated with flowering time are correlated with latitude [31]–[33], and in pines several genes contain variation that is correlated with temperature [34]. In addition, two recent studies that assayed hundreds of transposable elements in *Drosophila melanogaster* [35] and nearly 2000 SNPs in *Pinus taeda* [36] identified loci with evidence of selection related to climate.

In addition to correlations between allele frequencies and continuous climate variables, adaptations to different local environments can be identified by contrasting allele frequencies across populations classified based on categorical environmental variables, analyzed in a dichotomous manner. Studies of individual candidate genes have detected signals of correlation between allele frequencies and diet or mode of subsistence [37]–[38]. Recently, worldwide allele frequency data for SNPs on a genome-wide genotyping platform were analyzed to test for correlations between allele frequencies and categorical variables for main dietary component, mode of subsistence and ecoregion [39]. Though analyses of dichotomous variables are expected to be less powerful than tests of variables over a continuous range, this study found significant evidence at the genome-wide level for adaptations to a diet rich in roots and tubers, a foraging subsistence as well as polar, dry, and humid temperate ecoregions.

When assessing evidence for an ecocline, it is crucial to control for population history, which can pose several challenges for accurately assessing whether a correlation between a genetic variant and latitude or climate is due to natural selection [40]. For example, if migration patterns correspond closely with variation in a particular climate variable, the correlations between neutral alleles and that climate variable may be high even if selection has not acted on the locus. Conversely, if the effects of selection are subtle relative to the effects of population structure on allele frequencies, significance of correlations may be underestimated if population history is not taken into account. Using information about the background levels of variation in the genome, the relationships among populations can be modeled and the signal due to population structure can be taken into account.

Here, we use the same allele frequency data analyzed in Hancock et al (2010) [39] to test for adaptations to continuous climate variables at the genome-wide level and to identify genetic loci that underlie these adaptations. While several genome-wide scans for selection have been conducted in humans [41]–[47], only two used information about the environment to detect signatures of selection on a genome scale [39], [48]. However, these previous studies used less informative variables compared to those used here. Hancock et al. (2010) used dichotomous variables for the analysis and Fumagalli et al. (2010) used highly ascertained virus diversity data collected on a country-wide scale. Because the climate variables used here are continuous and are collected over a local scale, these analyses are expected to result in a more precise detection of selection signals. Further, since the continuous climate variables are only partially correlated with diet, subsistence and ecoregion, the present analysis detects new selection signals compared to those reported in Hancock et al (2010) [39] and those from other genome scans for selection. Importantly, while the adaptations to diet, subsistence and ecoregion tended to coincide with susceptibility SNPs for metabolic diseases and traits, the signals identified in this study show a different pattern, with a prominent role for pigmentation and immune response phenotypes. Therefore, through our approach, the impact of different selective pressures can be examined by testing for different (even if not completely independent) environmental variables.

## Results

**Figure 1:**
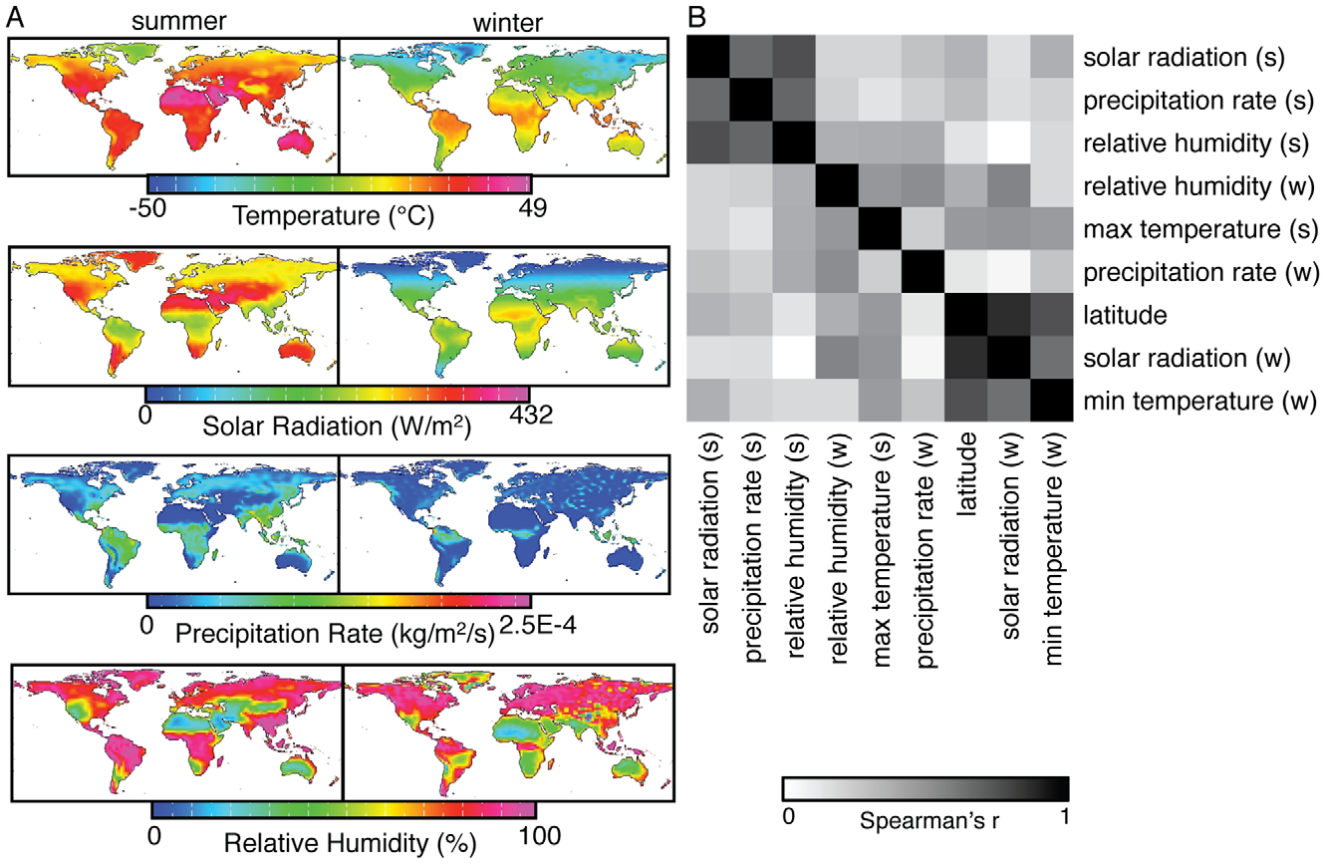
Climate variables used for the analysis. (A) Maps show the distributions of summer and winter climate variables: maximum summer temperature, minimum winter temperature and solar radiation, precipitation rate and relative humidity in the summer and winter. (B) A heatmap shows the absolute values of Spearman rank correlation coefficients between pairs of climate variables.

We analyzed genome-wide SNP data for 5 human populations genotyped by the Di Rienzo lab (Vasekela !Kung sampled in South Africa, lowland Amhara from Ethiopia, Naukan Yup'ik and Maritime Chukchee from Siberia, and Australian Aborigines Text S1) to complement publicly available data for the same SNPs in 52 Human Genome Diversity Project panel (HGDP) populations [49] and 4 HapMap Phase III populations (Luhya, Maasai, Tuscans and Gujarati) (www.hapmap.org). The 5 populations we genotyped are especially valuable because they expand information in Africa and Oceania where HGDP population coverage is low and they extend the range of environments to cover more extreme arctic climates. For each of the 61 populations, we gathered environmental data for nine continuous climate variables, chosen to capture those aspects of climatic variation that have a strong impact on human physiology (Figure 1). We note that these climate variables are meant as simple proxies for selective pressures that are likely more complex. Furthermore, the observed associations with particular climate variables may reflect selection for unrelated, but correlated, environmental pressures.

We assessed the evidence for a correlation between the allele frequency of each SNP and each environmental variable using a Bayesian linear model method that controls for the covariance of allele frequencies between populations due to population history and accounts for differences in sample sizes among populations [50]. Using a large set of randomly chosen SNPs, we estimated a covariance matrix of allele frequencies across populations. This covariance matrix forms the basis of the null model for the transformed allele frequencies at a SNP to be tested. Under the alternative model, this null model is augmented by a linear effect of an environmental variable.

At each SNP tested the method yields a Bayes factor (BF) as a measure of the support for the alternative model relative to the null model, in which the transformed population allele frequency distribution is dependent on population structure alone. In other words, the method asks whether selective pressures correlated with a climate variable have shaped spatial patterns of allele frequencies above and beyond the effect of population structure (as captured by the covariance matrix). As shown in Text S1, the population covariance matrices of the null model recover similar population clusters as those observed using other methods (e.g., STRUCTURE [49]–[52]) suggesting that our method captures the broad patterns of human population structure. It should be noted that the SNPs on the Illumina chip represent a biased subset of human diversity and this bias may affect measures of population differentiation [53]. However, while this may distort our estimate of the covariance matrix, it reflects the effect of population structure for the SNPs that we are testing, thus providing the correct adjustment for our test.

Although several SNPs had very large BFs with climate variables and might be considered “genome-wide significant” using general assumptions (see Methods, Text S2), BFs can be substantially inflated due to potential imperfections in the null model. Therefore, we used the BF only as a descriptive statistic to represent the strength of a correlation between each SNP and climate variable. In subsequent analyses, we ranked the SNPs based on their BFs to calculate a transformed rank statistic, with higher BFs corresponding to lower ranks (this transformed rank statistic is sometimes referred to as an ‘empirical p-value’). To account for possible differences in the distributions of the BFs across SNPs with different mean allele frequencies and ascertained using different schemes, we binned SNPs by global allele frequency and ascertainment panel (for a total of 30 separate bins). For each climate variable, each SNP was ranked relative only to the SNPs in the same bin; as a consequence, the lowest possible rank is in the order of 10^−5^.

We summarized the rank statistics for each SNP by calculating the minimum rank across all nine climate variables. To test for evidence of selection on the climate variables overall, we then calculated the proportion of SNPs likely to be enriched for functional effects (referred to as test SNPs) relative to the proportion of SNPs likely to be neutrally evolving (referred to as neutral SNPs) in the lower tail of the minimum rank distribution. In the absence of selection, equal proportions of these two classes of SNPs are expected to lie in the extreme tail of the BF distribution for any given cutoff. Conversely, if a higher portion of the test SNPs were targeted by selection than the neutral SNPs, an enrichment of test SNPs in the lower tail of the minimum rank distribution is expected. Conducting the analysis on the minimum rank statistic allowed us to assess the evidence of selection from the nine climate variables, overall. We also asked which of the individual climate variables were responsible for the signals we observed. For this analysis, we calculated the rank statistic for each SNP and each individual climate variable and, as in the previous analyses, we looked for an enrichment of large BFs among test compared to neutral SNPs. Finally, for individual SNPs that are discussed below, we quote the BF and the empirical rank specific to their ascertainment and mean frequency bin.

### Genic and nonsynonymous SNPs are enriched for signals of adaptations to climate

As shown in Table 1, there is an enrichment of test SNPs with large BFs relative to neutral SNPs; that is, the ratios of the proportions of both genic and nonsynonymous (NS) SNPs to the proportion of non-genic SNPs are significantly greater than 1 across three tail cutoffs (0.5%, 0.1% and 0.05%) of the minimum rank statistic distribution. Furthermore, the enrichment of genic and NS SNPs becomes progressively greater in the more extreme parts of the tail. Consistent with the fact that a larger fraction of NS SNPs compared to genic SNPs have functional effects, there is a greater enrichment of NS SNPs compared to genic SNPs in the tails of the distribution. These patterns suggest that the tail of the BF distribution contains true targets of positive selection. We next asked which individual variables were responsible for the enrichment of genic and NS SNPs observed in Table 1. As shown in Table 2, several individual climate variables exhibited strong signals, including latitude, solar radiation, relative humidity and temperature.

**Table 1:** Proportions of genic and nonsynonymous SNPs relative to the proportion of non-genic SNPs in the tail of the minimum rank distribution.

Variable category	Population set	Genic:Nongenic			NS:Nongenic		
		tail cutoff			tail cutoff		
		0.05	0.01	0.005	0.05	0.01	0.005
Climate	Worldwide	1.08***	1.14***	1.18***	1.25*	1.58***	1.63**
	AWE	1.06***	1.12***	1.12**	1.17**	1.37**	1.61*
	AEA	1.03	1.09**	1.17***	1.06	1.23	1.20

Symbols *, ** and *** denote support from >95%, >97.5% and >99% of bootstrap replicate, respectively.

**Table 2:** Proportions of genic and nonsynonymous SNPs relative to the proportion of non-genic SNPs in the tails of the individual variable distributions.

Season	Variable	genic:non-genic			NS:non-genic		
		tail cut-off:			tail cut-off:		
		0.05	0.01	0.005	0.05	0.01	0.005
	Latitude	1.07 ***	1.14***	1.19***	1.19***	1.60***	1.56***
summer	Maximum Temperature	1.02	1.06	1.13**	1.17**	1.33**	1.56***
	Precipitation Rate	1.00	1.02	1.02	1.03	1.14	1.24
	Relative Humidity	1.05**	1.20***	1.22***	1.06	1.21	1.40*
	Solar Radiation	1.05***	1.15***	1.16***	1.17**	1.35**	1.48**
winter	Minimum Temperature	1.04*	1.04	1.05	1.24***	1.37**	1.75***
	Precipitation Rate	1.04	1.12***	1.17***	1.10	1.23*	1.36
	Relative Humidity	1.07***	1.13***	1.14***	1.24***	1.20	1.24
	Solar Radiation	1.09***	1.08*	1.13*	1.26***	1.45***	1.24

Symbols *, ** and *** denote support from >95%, >97.5% and >99% of bootstrap replicate, respectively.

**Figure 2:**
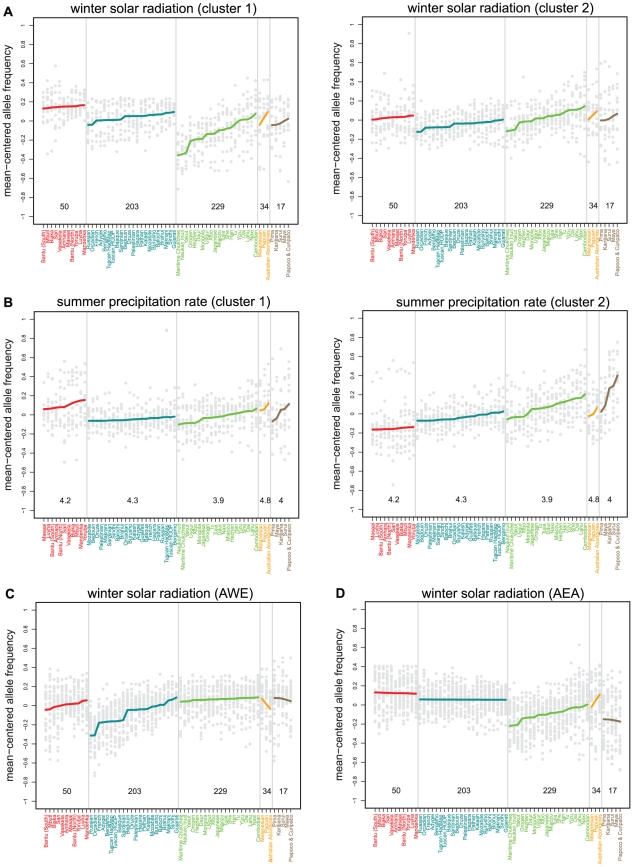
Mean-centered allele frequency plotted against population for SNPs with the strongest signals (transformed rank statistic <10^−5^). The variables shown are: (A) winter solar radiation in the worldwide analysis, (B) summer precipitation rate in the worldwide analysis, and winter solar radiation in (C) the AWE population subset and (D) the AEA population subset. Since the particular patterns that result in strong correlations in the worldwide analysis are diverse, SNPs for these variables were split into two clusters using the results of an eigen analysis of the matrix of SNPs and populations. SNPs were assigned to clusters based on the eigenvector term for the eigenvector corresponding to the first eigenvalue [91]. Mean-centered allele frequencies were computed by subtracting the mean allele frequency across populations. SNPs with rank statistics less than 10^−5^ are included in the plots. Population names and means are colored based on membership in one of five major geographical regions (sub-Saharan Africa, Western Eurasia, East Asia, Oceania, and the Americas) and ordered, within each region, so that the climate variable values increase from left to right across the x-axis. Alleles are polarized based on the signs of the Spearman correlations with the climate variable. Each gray dot represents an individual SNP and fitted lines (obtained using the lm function in R) for each region are shown in color. The ranges of the climate variable values across each geographic region are shown above the horizontal axis.

**Figure 3:**
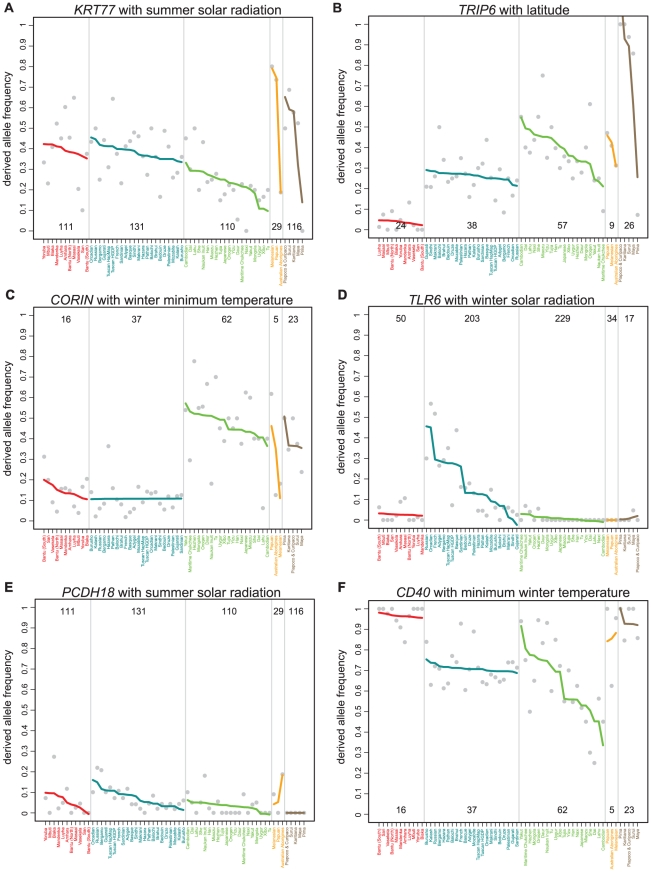
Global variation in allele frequencies for SNPs with strong signals with climate. Two NS SNPs from the worldwide analysis: (A) A SNP (rs3782489) in keratin 77 (*KRT77*), is strongly correlated with summer solar radiation, and (B) a SNP (rs2075756) in the thyroid receptor interacting protein (*TRIP6*) is strongly correlated with absolute latitude. Two SNPs from the population subset analysis: (C) A SNP (rs4558836) in *CORIN* has a signal in the AEA population subset with winter minimum temperature, but not in the AWE subset, and (D) a NS SNP (rs5743810) in TLR6 has a signal in the AWE population subset with winter solar radiation, but not in the AEA subset. Two SNPs that are associated with autoimmune disease from GWAS: (E) A SNP (rs2313132) upstream of *PCDH18* that is associated with SLE is strongly correlated with summer solar radiation, and (F) a SNP (rs6074022) upstream of *CD40* that is associated with multiple sclerosis is strongly correlated with minimum winter temperature. For each plot, gray points represent individual SNPs and colored lines represent fitted lines (obtained using the lm function in R) for each region. The ranges of the climate variable values for each region are shown at the bottom of the corresponding segment of the plot.

Figure 2A and 2B and Figure S1 show the patterns of allele frequency variation across populations for the SNPs with the strongest signals with each climate variable. We found that strong correlations at individual SNPs can result in diverse types of patterns. For example, while some signals appear to be driven by subtle, but consistent, changes in allele frequencies across regions (Figure 3A, 3B, 3E), others appear to be driven by only a subset of the regions (Figure 3F).

Among the strongest signals are several NS SNPs in genes that may play roles in heat and cold tolerance and in disease resistance. A SNP in keratin 77 (*KRT77*) (rs3782489), a gene that is specifically expressed in the ducts of eccrine sweat glands, is strongly correlated with summer solar radiation (log_10_BF = 9.12, rank statistic = 2.4×10^−5^), suggesting that this SNP may influence temperature homeostasis through sweating (Figure 3A). A SNP (rs2075756) in *TRIP6* has strong signals with absolute latitude (log_10_BF = 9.47, rank statistic = 1.4×10^−4^) and minimum winter temperature (log_10_BF = 11.6, rank statistic = 4.0×10^−4^) (Figure 3B). *TRIP6* interacts with LPA and thyroid hormones, proteins known to play a role in energy metabolism and basal metabolic rate [54], and implicated in the immune response to bacterial pathogens [55] and tumor invasiveness [56].

The plots in Figure 2A and 2B and Figure 3A, 3B, 3E show that the signals detected by our method consist of correlations between allele frequencies and climate variables that occur in parallel in multiple geographic regions, even though the slope of the correlation and the average allele frequency may vary across regions. These patterns indicate that human population structure can strongly influence the distribution of allele frequency of variants targeted by spatially-varying selective pressures. Furthermore, these patterns are reminiscent of the independent latitudinal clines observed in different hemispheres at the Adh locus in populations of *Drosophila melanogaster* [57]; these inverse clines are often considered the hallmark of climate-related selective pressures.

### Correlations with climate within geographically-restricted population subsets

Several recent studies have shown that convergent evolution underlies similarities in some phenotypes, e.g., skin pigmentation [58]–[59] and lactase persistence [60], across geographically separated populations, implying that similar environmental pressures can select different beneficial alleles in different populations. Therefore, SNPs selected in a geographically restricted subset of populations may be missed in the above analysis of worldwide populations. To improve our power of detecting this class of SNPs, we conducted the climate analysis within two population subsets: the populations in Africa and Western Eurasia (AWE), including the Middle East and West Asia, and the populations in Africa, East Asia and Oceania (AEA) (Text S3). These population subsets are suggested as natural groupings by previous work on both genome-wide and putatively selected loci in the HGDP [43], [52]. Plots of the strongest signals for each climate variable in the AEA and AWE subsets exhibit patterns that are indeed restricted to the specific regions included in each analysis (Figure 2C, 2D, Figures S2, S3, S4,).

Despite the smaller number of populations in each subset compared to the worldwide set, we find a significant enrichment overall for genic SNPs with strong correlations with climate relative to nongenic SNPs in all population subsets (Table 1). We found stronger evidence for selection in the AWE compared to the AEA population subset (even though the numbers of populations in the two subsets are similar). Several individual variables showed strong enrichment for genic and/or NS SNPs; the most striking were for the AWE population subset with relative humidity and solar radiation (Table S1).

There are several interesting genic and NS SNPs with strong signals in either the AEA or the AWE population subset. For example, several SNPs in the *CORIN* gene region (rs4558836, rs6447571, rs17601068) have strong signals with latitude and minimum winter temperature (log_10_BF = 21.9, 28.7 and 20.8 and rank statistics of 2.0×10^−5^, 3.1×10^−5^, and 2.1×10^−5^ with minimum temperature) in the AEA subset, but do not have strong signals with any climate variable in the AWE subset (Figure 3C). This gene has an important role in cardiovascular health because it encodes a protein that activates the precursor of natriuretic peptide, which in turn regulates blood volume and pressure. In addition, variation in *CORIN* may play a role in pigmentation; *CORIN* is an upstream regulator of agouti and variation in the gene in mice affects coloration (H. Hoekstra, personal communication). Three NS SNPs in the toll-like receptor gene cluster (*TLR1 N248S*, *TLR6 S249P* and *TLR10 N241H*) are strongly correlated with winter solar radiation in the AWE subset, but not in the AEA subset (log_10_BF = 9.0, 4.4, 10.2, and rank statistics = 3.0×10^−5^, 2.9×10^−5^, 3.6×10^−5^). At *TLR6 N249S* (rs5743810), which is associated with variation in resistance to malaria, the allele that confers resistance occurs at high frequency in Africa and Asia and lower frequency in European populations (Figure 3D). Population differentiation between European and South West Asian populations as measured by F_ST_ is also extremely high for this SNP [44].

### Concordance of signals between the population subset and the worldwide analyses

**Figure 4:**
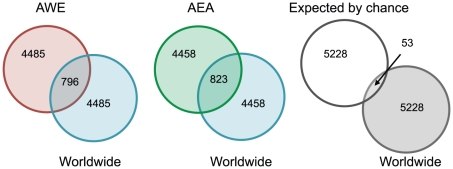
Venn diagrams showing the overlap between lower tails of rank statistics from the worldwide analysis and each population subset analysis. The Venn diagram on the right shows the overlap expected between the results of the worldwide analysis and a set of randomly drawn SNPs.

The majority of strong signals are specific to each population subset and do not overlap with those found in the worldwide analysis, suggesting that either the true selection pressures are more localized than our climate variable proxies or that many variants have undergone convergent evolution. However, there is also a strong enrichment of overlapping signals between each subset and the worldwide analysis compared to the overlap expected by chance (Figure 4, Text S3). Moreover, the minimum ranks in the AWE and AEA population subsets are weakly correlated (Spearman's rho = 0.19), while the correlation for each population subset and the worldwide analysis was substantially higher (Spearman's rho = 0.42 for AWE versus worldwide and Spearman's rho = 0.33 for AEA versus worldwide). These results indicate that some of the geographically restricted signals may be strong enough to be detected in both the subset and the worldwide analyses.

### Comparison to other studies of ecoclines

The analyses performed here are similar to a previous in-depth study of the energy metabolism pathway [25], but they also differ in several important respects. Specifically, the present study includes more populations (62 versus 54), has lower SNP density per gene, does not apply a minor allele frequency cutoff and uses a much larger number of SNPs as controls. In the previous study, we asked whether genes involved in energy metabolism *as a group* showed evidence of selection while in this study we test for evidence of selection at the genome-wide level. The inclusion of additional populations should increase the power to detect evidence of selection on this pathway, while the decreased SNP density and lack of a minor allele frequency cutoff should decrease power. It is hard to predict how the results are affected by the different set of control SNPs, although the larger number of control SNPs here is expected to result in a more accurate assessment of the relative strength of the signal.

To understand the effects of these differences, we compared the results from the two studies. First, we asked whether strong correlations with climate were enriched in the same energy metabolism gene set relative to other genic SNPs (using the same tail cutoffs we used for the tests of genic and NS SNP enrichment). We did not find a significant enrichment of signal for this gene set in this analysis (Table S2). To better understand what caused the difference, we conducted several additional analyses. We analyzed only the data for the subset of 52 populations that were included in both analyses, with and without the SNPs that were genotyped in Hancock et al (2008) [25]. There was no significant enrichment of signal for the energy metabolism gene set when only the Illumina 650Y SNPs were included; however, there was a significant enrichment of signal for several variables when the SNPs genotyped in Hancock 2008 were included, even though here the enrichment was assessed compared to a much larger set of genome-wide control SNPs [25] (Table S2). This suggests that the most important difference between this study and the previous one was the density of SNPs genotyped.

We also asked how our results compared to those from a recent analysis using the same populations and data, but different environmental variables (i.e. dichotomous variables that summarized information about ecoregion, diet and subsistence). We found significant, but weak correlations between the results from this analysis and the previous one (Pearson's correlation coefficients range from −0.001 (between average maximum temperature in the summer and a horticultural subsistence pattern) to 0.3 (between relative humidity in the summer and dry ecoregion)) and that the majority of the strongest signals differed across tests (Figures S5 and S6). We also compared our results to the top 30 regions identified in a scan for correlations between SNP allele frequency and virus diversity [48], but did not find any overlap in the extreme tail. The strongest climate transformed rank statistic for any variable with virus diversity was 0.002 for a SNP (rs4852988) in Annexin IV with solar radiation in the summer. This gene is involved in the NF-kappaB signaling pathway [61] and is implicated in renal and ovarian clear cell carcinoma [62]–[64].

### Overlap with results from GWAS

Results of genome-wide association studies with diseases and other complex traits offer an opportunity to connect signals of selection with SNPs influencing specific traits and diseases. To this end, we identified a subset of SNPs with extremely large BFs for climate variables that were also strongly associated with traits based on the results of 106 GWAS (Table 3). Among the SNPs that were strongly correlated with climate, several are implicated in pigmentation and autoimmune disease. Signals with pigmentation appear to be driven mainly by patterns in the AWE subset, possibly reflecting the fact that most GWAS studies were conducted in European populations (Figure S7). Figure S7 shows variation in allele frequencies versus solar radiation for two SNPs that are strongly associated with pigmentation in the AWE population subset: a SNP in *SLC45A2* (rs28777) (log_10_BF = 10.4, rank statistic = 4.2×10^−5^) that is associated with hair color and a SNP in *OCA2* (rs1667394) (log_10_BF = 8.3, rank statistic = 5.0×10^−5^) that is associated with eye and hair color. In addition, consistent with the notion that pathogens exerted powerful selective pressures on humans [65], we observed strong signals of selection for several variants that are associated with diseases of the immune response. Specifically, these signals include: for the worldwide analysis, SNPs in or near *PCDH18* (Figure 4A), *PTGER4* and *CD40* (Figure 3E, 3F) that are implicated in systemic lupus erythematosus (SLE), Crohn's disease and multiple sclerosis, and for the population subset analyses, SNPs in or near *HLA-DQ1*, *CD40*, *HLA-C*, *IL13* and *UBASH3A* that are associated with SLE, celiac disease, multiple sclerosis, psoriasis and type 1 diabetes showed signals.

**Table 3:** SNPs with the strongest signals of selection among those associated with phenotypic traits in GWAS.

Trait category	Strongest disease or trait association	Ref SNP ID	Most significant climate correlation				Nearby genes
			Pop Set	Variable	log_10_BF	Rank Statistic	
Pigmentation and tanning	Hair Color	rs12913832	WW	Summer Maximum Temperature	7.06	2.08×10^−5^	*HERC2*
	Hair Color	rs12913832	WW	Summer Relative Humidity	8.11	2.08×10^−5^	*HERC2*
	Hair Color	rs28777	AWE	Winter Solar Radiation	10.4	4.22×10^−5^	*SLC45A2*
	Hair Color	rs28777	WW	Winter Relative Humidity	4.26	3.29×10^−4^	*SLC45A2*
	Eye Color	rs1667394	AWE	Winter Solar Radiation	8.33	4.99×10^−5^	*OCA2*
	Hair Color	rs1667394	AWE	Winter Solar Radiation	8.33	4.99×10^−5^	*OCA2*
	Tanning	rs35391	WW	Summer Relative Humidity	7.27	6.81×10^−5^	*SLC45A2*
	Tanning	rs35391	AWE	Winter Solar Radiation	6.63	3.50×10^−4^	*SLC45A2*
Immune and Autoimmune	Multiple sclerosis	rs6074022	AEA	Summer Precipitation Rate	6.98	4.00×10^−4^	*CD40*
	Multiple sclerosis	rs6074022	WW	Winter Minimum Temperature	11.1	2.40×10^−4^	*CD40*
	SLE	rs2313132	WW	Summer Solar Radiation	2.05	4.52×10^−4^	*PCDH18*
	SLE	rs2187668	AWE	Summer Relative Humidity	8.25	1.82×10^−5^	*HLA-DQA1*
	Celiac Disease	rs2187668	AWE	Summer Relative Humidity	8.25	1.82×10^−5^	*HLA-DQA1*
	Crohn's disease	rs4613763	WW	Summer Relative Humidity	2.19	2.26×10^−4^	*PTGER4*
	Psoriasis	rs10484554	AEA	Summer Precipitation Rate	7.23	1.80×10^−4^	*HLA-C*
	AIDS progression	rs10484554	AEA	Summer Precipitation Rate	7.23	1.80×10^−4^	*HLA-C*
Height	Height	rs185819	AEA	Summer Maximum Temperature	5.55	4.79×10^−4^	*TNXB (HLA class III)*
Cardiovascular	Stroke	rs10486776	AWE	Winter Solar Radiation	2.3	2.94×10^−4^	*MEOX2*
	Factor VII	rs10488360	AWE	Summer Precipitation Rate	6.76	2.06×10^−4^	*SDK1*
Other	Bone Mineral Density (Hip)	rs10490823	AWE	Winter Solar Radiation	5.53	4.54×10^−4^	*CTNNB1*
Other	Testicular germ cell tumor	rs210138	AEA	Summer Precipitation Rate	8.14	1.50×10^−4^	*BAK1*

This table contains SNPs with an empirical rank less than 5×10^−4^ and a GWAS p-value of less than 1×10^−5^.

### Enrichment of signal in sets of genes

To learn about the biological pathways that were targeted by selection, we asked whether there is an enrichment of signal for particular sets of genes using three classifications: genes associated with major disease classes, genes in canonical pathways, and genes that are up or down-regulated in response to chemical or genetic perturbations. Because proportionally more genic than non-genic SNPs have strong correlations with climate variables, an enrichment of signals for SNPs in a particular gene set relative to non-genic SNPs may simply reflect the global genic enrichment. Therefore, in this analysis, we tested whether the proportion of genic SNPs from a given set was greater than the proportion of genic SNPs from other genes not in that set, within the tail of the rank statistic distribution.

In the disease class analysis, the strongest signals were with cardiovascular and immune diseases (Table 4). Overall, the disease classes showed a much greater concentration of signals compared to the other two classifications. Of the 14 disease classes tested, 7 (50%) showed signals in at least one analysis. This was remarkable compared to the proportions observed for either the canonical pathways (0.025%) or chemical and genetic perturbations (0.033%). This difference might be explained by the fact that genes in canonical pathways and differentially expressed sets, while biologically important, may not contain segregating functional variation.

**Table 4:** Disease classes enriched in the 1% and 5% tails of the minimum rank distribution.

Variable	Geographic Region	Disease Class	SNPs in gene set: other genic SNPs		
			tail cutoff:		
0.05	0.01	0.005
Climate	Worldwide	Cardiovascular	1.27***	1.45*	1.69*
	AWE	Cancer	1.24**	1.54***	1.76**
		Cardiovascular	1.39***	1.49***	1.50*
		Immune	1.33***	1.58***	1.85***
		Infection	1.32*	1.65**	2.19**
	AEA	Cardiovascular	1.25**	1.69**	2.05***
		Immune	1.14*	1.51*	1.88**

Symbols *, ** and *** denote support from >95%, >97.5% and >99% of bootstrap replicate, respectively.

Several of the signals for canonical pathways and differentially expressed gene sets are also worth noting (Table 5 and Table S3). Two long-standing hypotheses [16], [66] state that solar radiation and temperature have been important selective forces among human populations, and these hypotheses have gained population genetic support from several previous studies [22], [24]–[25], [44], [58]–[59]. Accordingly, we find an enrichment of strong correlations with climate variables for gene sets that are differentially regulated in response to UV radiation and genes that are central in the differentiation of brown adipocytes, a tissue that plays an important role in cold tolerance through non-shivering thermogenesis. Consistent with our findings in the GWAS overlap analysis and in the disease class analysis, we identified several gene sets that are related to immunity and inflammation. Interestingly, we also identified a large number of climate signals in genes related to breast, prostate and colon cancer, three types of cancers with significant disparities among US populations [67]. Given the observed links between cancer and inflammation [68], one potential explanation for this finding is that genetic variation that enhances the immune response to pathogens may result in increased susceptibility to cancer.

**Table 5:** A subset of the strongest results for chemical and genetic perturbations.

Related Trait/Disease	Pop Set	Description	SNPs in gene set: other genic SNPs		
			cutoff:		
			0.05	0.01	0.005
Response to UV radiation	AEA	Down-regulated at 6 hours following treatment of WS1 human skin fibroblasts with UVC at a low dose (10 J/m∧2)	2.37**	4.56***	4.58*
	AEA	Down-regulated at any time-point following treatment of both XPB/CS and XPB/TTD fibroblasts with 3 J/m∧2 UVC	1.22*	1.57**	1.65*
	AEA	Down-regulated at 8 hours following treatment of XPB/CS fibroblasts with 3 J/m∧2 UVC	1.18***	1.43**	1.63***
	AEA	Down-regulated at any time-point following treatment of XPB/CS fibroblasts with 3 J/m∧2 UVC	1.16**	1.41**	1.57***
Thermo-regulation	AEA	Up-regulated in brown preadipocytes from Irs1-knockout mice, which display severe defects in adipocyte differentiation	1.43**	1.60*	2.07*
Cancer/Cell proliferation	WW	Down-regulated with stable, ectopic overexpression of BRCA1 in human prostate cancer cell lines	2.01***	3.24***	4.31**
	WW	Genes concomitantly modulated by activated Notch1 in mouse and human primary keratinocytes	1.40*	1.90*	2.50**
	WW	Genes up-regulated in kras knockdown vs control in a human cell line	1.46***	2.08***	2.74***
	AEA	Genes up-regulated in kras knockdown vs control in a human cell line	1.34***	2.11***	2.24***
	AEA	Gene set that can be used to differentiate *BRCA1*-linked and *BRCA2*-linked breast cancers	1.48**	2.45**	3.49**
	AEA	Up-regulated by butyrate at 24 hrs in SW260 colon carcinoma cells	1.61**	2.30**	2.88**
	AWE	Up-regulated by sulindac at 48 hrs in SW260 colon carcinoma cells	1.45**	2.10*	2.52*
	AWE	Genes up-regulated by NF-kappa B	1.30*	1.96*	2.37**
	AWE	Down-regulated in cells undergoing IL-3-dependent proliferative self-renewal	1.54***	2.47***	2.74***
	AWE	Up-regulated in human dermal (foreskin) microvascular endothelial cells that were stimulated to proliferate with prolonged EGF treatment	1.81**	2.88**	5.34***
Infection/Immunity	AWE	Genes up-regulated by NF-kappa B	1.30*	1.96*	2.37**
	AEA	Down-regulated in fibroblasts following infection with human cytomegalovirus	2.20*	3.59*	6.03**
	AEA	Genes down-reglated in peripheral blood lymphocytes (PBLs) of immunosuppressed patients with a well functioning kidney transplant	1.84***	3.37**	5.10**
	AWE	Down-regulated in cells undergoing IL-3-dependent proliferative self-renewal	1.54***	2.47***	2.74***
	AWE	Up-regulated in human dermal (foreskin) microvascular endothelial cells that were stimulated to proliferate with prolonged EGF treatment	1.81**	2.88**	5.34***
	AWE	Genes up-regulated in peripheral blood lymphocytes (PBLs) of stable, immunosuppressed patients with a well functioning kidney transplant	1.74***	2.29***	2.58**

## Discussion

Here, we presented the results of a genome-wide scan for evidence of positive selection in response to climatic variation. Climate is known to influence the distribution of human pathogens [69]. Accordingly, many of our signals coincide with SNPs associated with diseases of the immune response in GWAS studies. Therefore, it is likely that our analysis detects the action of selective pressures that are due to climate or are broadly mediated by climate. In this study, we carefully controlled for the effects of population history in two ways. First, we used a null model for the covariance of allele frequencies across populations, estimated based on genome-wide SNP data. Second, we assessed the evidence for selection in terms of a transformed rank statistic; in other words, we used genomic controls to detect SNPs with the strongest genome-wide signals of selection. Unlike haplotype and frequency spectrum based approaches to detect selection, our method does not assume a model in which a new variant was driven quickly to high frequency in the population. Indeed, many of our strongest findings are for alleles that exhibit correlations between allele frequency and climate variables in parallel in multiple continental regions, suggesting that selection acted on standing alleles with a broad geographic distribution. It has been argued that selection on standing variation played an important role in the adaptive evolution of complex traits in humans [70]–[71]; therefore, the signals that we detected may help elucidate the genetic architecture of common phenotypes with complex patterns of inheritance. As expected based on the use of climate variables to detect the impact of selection, the strongest signals in our analysis tend to differ from SNPs that show extreme patterns in F_ST_ or haplotype homozygosity-based analyses. When we compared our results to the results of a global F_ST_ analysis for the AWE and AEA subsets, we found only a slight excess of overlap in the 5% tail compared to that expected by chance (1.36 and 1.11 fold) and no excess of overlap with the results from an analysis using the integrated haplotype score (iHS) [72] (see Hancock et al. [70] for a more extensive discussion). In addition, we find little overlap between the signals found in this analysis, which uses climate variables, and those found, in the same data, for environmental variables related to diet, subsistence and ecoregion [39] or with the strongest signals from a study that examined virus diversity [48].

Climate is known to have an important impact on animal physiology and fitness, as a result of both direct and indirect effects. Direct effects include heat and cold stress, dehydration stress, and stress resulting from too much or too little UV radiation. For example, variation in temperature and relative humidity can result in cold or heat stress, i.e. a deviation from the relatively narrow range of body temperatures that is optimal for the coordination of molecular and cellular processes. Likewise, variation in exposure to solar radiation influences vitamin D production in the skin [18] and the breakdown of folate [73], both with important consequences for human health [74]. Protracted exposure to extreme temperatures, as it occurs during heat waves, results in heat exhaustion and heat stroke, and is associated with increased mortality in the elderly and in children [75]; likewise, heat stress has an important influence on birth weight [76] and, as a consequence, on infant mortality. Variation in human morphology, including body size and shape, follows from basic thermoregulatory principles, to dissipate or conserve body heat in different climates. Metabolic adaptations are also observed in populations living in cold climates [77]. Moreover, extensive variation in heat and cold tolerance is reported across populations living in different climates (reviewed in Beall and Steegman [78]). Climate can also affect human physiology indirectly through its effects on the environment that humans live in. Among these effects, perhaps the most important one from the evolutionary standpoint is the role of climate in shaping the geographic distribution of human pathogens, with variation in precipitation rate being the best predictor of pathogen species diversity [69]. Although there is extensive evidence for phenotypic adaptations to different climates, the extent to which this variation is the result of genetic adaptations rather than developmental plasticity and acclimatization is unclear. Although it was previously shown that genetic adaptations to different climates had occurred in the gene network underlying common metabolic disorders [25], these results provide strong evidence for a wide variety of genetic adaptations to different climates at the genome-wide level. Moreover, the gene set analyses point to biological pathways, e.g., genes up or down-regulated in response to UV radiation and genes up-regulated in brown pre-adipocytes during differentiation, that are consistent with the impact of UV and cold stress on human physiology and evolutionary fitness. Furthermore, we find evidence for selection on loci involved in temperature homeostasis and immune response, based on overlap between individual signals of selection due to climate and loci associated with phenotypes in GWAS studies. Finally, it should be emphasized that some of the signals identified in this survey may be due to selective pressures that are correlated with climate variables, but are not due to either the direct or indirect effects of climate. This may be a specific concern for the population subset analyses where we test for parallel ecoclines across a smaller set of geographic regions.

GWAS have helped to clarify the genetic underpinnings of many disease phenotypes, but there are still many outstanding questions. Most human genes appear to be under strong purifying selection [79]–[80]. However, a sizable fraction of disease risk variants seem to be present at appreciable frequencies. Therefore, it has been hypothesized that these disease risk variants are either selectively neutral or that they have been acted on by positive selection [81]. Here, we reported evidence for selection at several individual SNPs identified by GWAS, on sets of genes implicated in cardiovascular and immune diseases, and on sets of differentially genes in response to chemical and genetic perturbations. Common themes that emerged from these disparate analyses are that genes and variants implicated in pigmentation and response to UV radiation, immune response, autoimmune disease and cancer are among those with the strongest signals of selection. In some of these cases, other factors that are influenced by and therefore correlated with climate (e.g. pathogen distribution or diet) are likely to be responsible for the observed signal. This is especially likely to be the case for variants implicated in immune response because pathogen distributions are influenced by climate [69]. Therefore, our results complement previous analyses that assess evidence for correlations with diet [39] and viral diversity [48]. However, since available measures of climate-correlated pathogens or diet are incomplete and possibly inaccurate, using a climate proxy may afford as much or even greater power to detect signals of selection.

Our results suggest that many loci that appear to be under selection due to climate may yield both positive and negative effects on fitness, and may therefore fit an antagonistic pleiotropy model [82]. According to this model, the negative pleiotropic effects of alleles that confer a fitness advantage early in life may contribute to the prevalence of diseases and disorders that tend to occur later in life. The selection signals that are related to immunity, in particular, may implicate variants evolving under a model of antagonistic pleiotropy. In these cases, the positive fitness effects of pathogen resistance may outweigh negative consequences of inflammatory processes, such as autoimmune disorders and cancer. For example, we found very strong evidence of selection for eight GWAS SNPs implicated in autoimmune disorders. In these cases, it is likely that the selective pressure was pathogen resistance, and that the autoimmune disorder is a pleiotropic consequence of the resistance allele. In addition, we identified an extremely strong signature of selection for *TLR6* P249S (rs5743810). The haplotype containing this SNP was previously found to show evidence of positive selection [44], [83] and is implicated in both malaria resistance and prostate cancer risk. Further, we found evidence for a concentration of signals of selection in gene sets implicated in cancer and immune function/inflammation from the disease class and expression gene set analysis. One especially interesting signal is for genes up-regulated by NF-kappa-B, because this transcriptional response is likely to mediate the association between inflammation and tumor progression [68], [84]. Taken together, these results suggest that positive selection acted on variants that enhance the immune and inflammatory response to pathogens, but that also increase risk for disease phenotypes such as autoimmune diseases and cancer.

The results of this genome scan not only increase our understanding of the genetic landscape of adaptation across the human genome, but they may also have a more practical value. For example, they can be used to select candidate genes for common disease risk and to generate specific testable hypotheses regarding the functions of specific genes and variants. While the results of genome-wide scans for association with diseases and other traits are accumulating at a rapid pace, interpretation of these results is often ambiguous because the power to detect all common variants that are important in the etiology of the phenotype is incomplete. This is especially true in the case of complex traits, where variants at many loci may contribute to the phenotype, each with a small effect. By combining the evidence from GWAS with evidence of selection, it may be possible to separate true causative regions from the background noise inherent in genome-wide screens for association. To facilitate this, all of our empirical rank statistics are publically available. Moreover, results of selection scans that detect evidence for spatially-varying selection may be especially relevant to diseases that show substantial differences in prevalence across ethnic groups (e.g., sodium-sensitive hypertension, type 2 diabetes, prostate cancer, osteoporosis). In the future, this approach could be extended by including additional populations and aspects of the environment to gain a more complete understanding of how natural selection has shaped variation across the genome in worldwide populations. Furthermore, whereas we relied on linkage disequilibrium between (potentially un-genotyped) adaptive variants and genotyped SNPs, whole genome re-sequencing data should give a more complete picture of the variation that underlies adaptation.

## Methods

### Populations included in the analysis

We used data from 61 worldwide human populations (Text S1), including 938 unrelated individuals from 52 Human Genome Diversity Project panel populations previously genotyped by Li et al. [49], 4 HapMap phase 3 populations, including 71 Luhya of Webuye, Kenya, 61 Maasai of Kinyawa, Kenya, 77 Tuscans in Italy, and 79 Gujarati Indians from the Gujarat Province, India, but collected in Houston, Texas and 5 populations genotyped by our group. Individuals genotyped by our group include 22 Vasakela !Kung from Angola, but whose samples were collected in Schmidtsdrift, South Africa, 22 Amhara residing at low altitude in northern Ethiopia, 22 Naukan Yup'ik and 22 Maritime Chukchee from Siberia and 8 Australian Aborigines from the European Collection of Cell Cultures. Although some of the five populations genotyped in our lab show some evidence of recent admixture (data not shown), they are included because they extend the geographic range of our analysis to regions that have no coverage in current collections of population samples. Moreover, despite the evidence for admixture, these populations still cluster as expected based on genetic affinities of populations (Text S1). Finally, the inclusion of admixed groups is expected to dampen the signal of local adaptation based on allele frequency differences, in general, and therefore should not lead to an increase in the false positive rate.

### SNP data collection

Illumina Infinium HumanHap 650Y genotype data for HGDP panel were obtained from Li et al. [49]. Additional populations were genotyped using the same type of genotyping chips at UCLA Southern California Genotyping Consortium Facility. HapMap phase 3 draft 1 (released 09/24/2009) genotype data were obtained from the HapMap Consortium website (http://www.hapmap.org/). Total numbers of SNPs and number of genic, non-genic and nonsynonymous SNPs included in the final analysis are shown in Table S4.

### Climate variables

Climate data were obtained for each population based on the coordinates of the locations where the samples were collected, except for the Vasakela !Kung and the Gujarati, who had recently relocated. For these populations, we used approximate coordinates of their homelands. The individuals who were sampled from the !Kung population were known to have recently relocated to Schmidtsdrift, South Africa from the Angola/Namibia border, and the Gujarati are originally from Gujarat, India. We selected climate variables separately for the summer and winter seasons from the NCEP/NCAR database [85] and obtained values for each variable by averaging over the three-month periods. Variables included in the final analyses were summer and winter precipitation rate, relative humidity, solar radiation, summer maximum temperature and winter minimum temperature. We also included latitude in this analysis. Global distributions of variables and correlations among variables are shown in Figure 1.

### Correlations between allele frequencies and climate variables

To calculate correlations between SNP allele frequencies and climate variables, we used a Bayesian linear model method that controls for population history by incorporating a covariance matrix of populations and that accounts for differences in sample size among populations [50]. This method yields a Bayes factor (BF) that is a measure of the weight of the evidence for a model in which an environmental variable has an effect on the distribution of the variant relative to a model in which the environmental variable has no effect on the distribution of the variant. In the case where the null model (i.e., the covariance matrix of populations) fully accounts for population structure, a suitable cutoff for genome-wide significance of a SNP-climate log_10_BF is 6.36, which, under general assumptions, is equivalent to a genome wide significant p-value of 8.5×10^−9^, or a p-value of 5% divided by 650,000 tests across 9 climate variables [86]–[87]. However, our null model, while flexible, necessarily makes simplifying assumptions, e.g., we approximate the correlated levels of genetic drift across populations by a multivariate normal distribution. Since we cannot expect the null model to account fully for the effects of population structure, we emphasize that we cannot take the BFs themselves at face value, nor can they be directly compared across climate variables. Therefore, we took a conservative approach and conducted subsequent analyses by comparing each SNP to the empirical distribution. To this end, for each SNP and each environmental variable, we calculated a transformed rank statistic (sometimes referred to as an “empirical p-value”) that was scaled to be between 0 and 1 (with 0 and 1 corresponding to the highest and lowest BF, respectively).

The Illumina genotyping array contains SNPs that were chosen to be tagging SNPs; in addition, they were subjected to ascertainment schemes that differ across the three major subsets of SNPs on the array [88]. To account for differences in ascertainment across SNP subsets within the array, we performed all analyses (including the estimation of the covariance matrix for the null model) separately in each subset. In addition, to account for allele frequency differences, the SNPs within each subset were separated into 10 bins based on their derived allele frequency in the global population sample. Within each bin and each SNP subset, SNPs were ranked according to their BFs. Then, for each SNP and environmental variable, we calculated the transformed rank statistic. Due to the complexity of the ascertainment protocol, we cannot rule out the possibility that some biases were not completely corrected for in our analyses.

To reduce the number of tests while assessing the strength of the evidence for selection with climate as a whole, we also calculated the minimum of the transformed rank statistics across all variables for each SNP. We used the rank and minimum rank statistics to look for an enrichment of large BFs (low rank statistics) in one group of SNPs compared to another.

### Assessing the evidence for an excess of genic and nonsynonymous SNPs in the tail of the distribution

To determine whether there was an excess of SNPs with strong signals that are enriched for functional variation, we compared the proportions of nonsynonymous (NS) and genic SNPs in the lower tail of the rank statistic distribution to the proportion of non-genic SNPs in this tail. Given the arbitrary nature of choosing a single cutoff, we set three cutoffs (5%, 1% and 0.5%). In other words, we looked at the top 5%, 1% and 0.5% of all BFs and asked whether there is an enrichment of genic and NS SNPs for each tail cut-off. A value of 1 represents no excess and a value greater than 1 represents an enrichment in the tail of the distribution. SNPs below the cutoff are likely to cluster along the genome due to linkage disequilibrium, thus reducing the number of independent signals contributing to an observed enrichment. To account for this possibility, we found the confidence interval for the enrichment using a bootstrap approach. To this end, we separated the genome into 500kb segments and then we bootstrap resampled a number of segments equal to the length of the genome divided by 500 kb. Just as we did for the observed data, for each of 1000 bootstrap replicates, we calculated the ratios of the relative proportions of genic and NS SNPs to the proportion of non-genic “neutral” SNPs in the tail of the minimum rank distribution. In this and other analyses described here, we considered an enrichment significant (with a one-tailed test) if at least 95% of the bootstrap replicates were enriched (e.g., had a ratio above 1).

### Population subset analysis

We calculated correlations between each SNP and each climate variable for geographically defined subsets of populations using the same Bayesian linear model method we used for the worldwide populations. For these analyses, we re-estimated the covariance matrices for each of the population subsets and for each SNP ascertainment panel. We defined two population subsets. One subset included the populations in sub-Saharan Africa as well as the populations in the Middle East and Western Eurasia (AWE) and the other included populations in sub-Saharan Africa and the populations in East Asia and Oceania (AEA). Populations included in each subset are shown in Text S3. We calculated rank statistics and minimum rank statistics for the population subset BFs and conducted all downstream analyses of enrichment for categories of SNPs and gene sets using the same methods described for the worldwide analysis. In addition, we found SNPs that had strong signals in both GWAS and the climate analysis as described above.

### Comparison to GWAS results

We downloaded the information in the Catalog of Published Genome-Wide Association Studies [89] on July 14, 2009, which lists reported SNP-trait associations with p-values less than 1tm10^−5^. This database contained entries for 800 unique autosomal SNPs found on the Illumina HumanHap650Y platform and 61 traits. From this list of SNPs, we identified a set of SNPs with extremely low rank statistics (less than 5×10^−4^) for each of the nine climate variables included in our analyses. Because most GWAS are performed in populations of European ancestry, the SNPs in the Illumina panel were binned based on the allele frequency in Europeans rather than the global allele frequency.

### Gene sets included in the analysis

Lists of genes implicated in major classes of disease were obtained from the Genetic Association Database (http://www.ncbi.nlm.nih.gov/dbGaP) [90]. This database contains information, mainly from candidate gene studies, about variants that were previously associated with specific diseases and classes of disease. We downloaded the entire database and created 14 gene sets comprised of genes that contained variants that were significantly associated with each disease class. Disease classes included aging, cancer, cardiovascular, developmental, hematological, immune, infection, metabolic, neurological, pharmacogenetic, psychiatric, renal, reproduction, and vision.

From the Molecular Signatures Database (http://www.broadinstitute.org/gsea/msigdb), we obtained 438 sets of genes involved in canonical pathways and 1168 sets of genes that were up or down-regulated in response to chemical or genetic perturbations.

### Gene set analysis

To determine whether there was an enrichment of signal for each gene set, we compared the proportion of SNPs from a given gene set to the proportion of all other genic SNPs in the tail of the minimum rank distribution and of the transformed rank distributions for the individual variables with the strongest genic enrichment. To assess the significance for the findings, and to ensure that the results were not driven by one or a few genomic regions, we applied the same bootstrap approach described above.

### Data access and availability

The results of these analyses will be made available through a searchable online data base, dbCLINE (http://genapps.uchicago.edu/dbcline), and linked to the HGDP Selection Browser (http://hgdp.uchicago.edu/cgi-bin/gbrowse/HGDP) to allow for the comparison of selection signals from different types of analyses.

## Supporting Information

Figure S1 Transformed allele frequency plotted against each of seven climate variables for SNPs with the strongest signals in the worldwide analysis. Since the particular patterns that result in strong correlations in the worldwide analysis are diverse, SNPs for these variables were split into two clusters using the results of an eigen analysis of the matrix of SNPs and populations. SNPs were assigned to clusters based on the eigenvector term for the eigenvector corresponding to the first eigenvalue. Panels include: (A) absolute latitude, (B) maximum summer temperature, (C) minimum winter temperature, (D) winter precipitation rate, (E) summer solar radiation, (F) summer relative humidity, and (G) winter relative humidity. Transformed allele frequencies were computed by subtracting the mean allele frequency across populations. SNPs with rank statistics less than 1e-5 are included in the plots. Population names and means are colored based on membership in one of seven major geographical regions (sub-Saharan Africa, Europe, Middle East, West Asia, East Asia, Oceania, or the Americas) and ordered so that the climate variable values increase from left to right across the x-axis. Each gray dot represents an individual SNP and fitted lines for each region are shown in color. The range of each climate variable across the geographic region is shown in each section of the plot.
(6.30 MB TIF)

Figure S2 Transformed allele frequency plotted against each of eight climate variables for SNPs with the strongest signatures of selection in the AWE population subset. Panels include: (A) absolute latitude, (B) maximum summer temperature, (C) minimum winter temperature, (D) summer precipitation rate, (E) winter precipitation rate, (F) summer solar radiation, (G) summer relative humidity, and (H) winter relative humidity. Transformed allele frequencies were computed by subtracting the mean allele frequency across populations. SNPs with rank statistics less than 1e-5 are included in the plots. Population names and means are colored based on membership in one of seven major geographical regions (sub-Saharan Africa, Europe, Middle East, West Asia, East Asia, Oceania, or the Americas) and ordered so that the climate variable values increase from left to right across the x-axis. Each gray dot represents an individual SNP and fitted lines for each region are shown in color. The range of each climate variable across the geographic region is shown in each section of the plot.
(4.50 MB TIF)

Figure S3 Transformed allele frequency plotted against each of eight climate variables for SNPs with the strongest signatures of selection in the AEA population subset. Panels include: (A) absolute latitude, (B) summer maximum temperature, (C) winter minimum temperature, (D) summer precipitation rate, (E) winter precipitation rate, (F) summer solar radiation, (G) summer relative humidity, and (H) winter relative humidity. Transformed allele frequencies were computed by subtracting the mean allele frequency across populations. SNPs with rank statistics less than 1e-5 are included in the plots. Population names and means are colored based on membership in one of seven major geographical regions (sub-Saharan Africa, Europe, Middle East, West Asia, East Asia, Oceania, or the Americas) and ordered so that the climate variable values increase from left to right across the x-axis. Each gray dot represents an individual SNP and fitted lines for each region are shown in color. The range of each climate variable across the geographic region is shown in each section of the plot.
(4.50 MB TIF)

Figure S4 Two SNPs implicated in pigmentation phenotypes that have strong correlations with winter solar radiation in the AWE population subset. (A) rs1667394, a SNP in OCA2, and (B) rs28777, a SNP in SLC45A2.
(3.00 MB TIF)

Figure S5 SNPs with transformed rank statistics less than 10-4 with any climate variable are listed in the figure. For each SNP, the strength of the transformed rank statistic (TRS) with all climate variables from this analysis as well as all ecoregion, diet and subsistence variables from the previously published Hancock 2010 analysis are shown using color-coding. Red represents a TRS < 1e-5, dark orange represents a TRS < 1e-4, orange represents a TRS < 1e-3, dark yellow represents a TRS < 1e-2 and light yellow represents a TRS < 1e-1. SNPs that were not analyzed in the previous study are colored gray. All other SNP-environmental variable combinations are colored white.
(0.43 MB EPS)

Figure S6 SNPs with transformed rank statistics less than 10-5 with any climate variable are listed in the figure. For each SNP, the strength of the transformed rank statistic (TRS) with all climate variables from this analysis as well as all ecoregion, diet and subsistence variables from the previously published Hancock 2010 analysis are shown using color-coding. Red represents a TRS < 1e-5, dark orange represents a TRS < 1e-4, orange represents a TRS < 1e-3, dark yellow represents a TRS < 1e-2 and light yellow represents a TRS < 1e-1. SNPs that were not analyzed in the previous study are colored gray. All other SNP-environmental variable combinations are colored white.
(0.18 MB EPS)

Figure S7 Signals for SNPs implicated in pigmentation and tanning in the worldwide, AWE and AEA analyses.
(0.59 MB EPS)

Table S1 Proportion of genic relative to nongenic and nonsynonymous relative to nongenic SNPs in the tails of the minimum rank distribution for population subset analysis with individual climate variables. Symbols *, ** and *** denote support from >95%, >97.5% and >99% of bootstrap replicate, respectively.
(0.02 MB XLS)

Table S2 Reanalysis of enrichment of correlations with climate for SNPs in an energy metabolism gene set (first published in Hancock et al., 2008)[25] compared to other genic SNPs.
(0.03 MB XLS)

Table S3 Canonical pathways and sets of genes differentially expressed in response to chemical and genetic perturbations enriched in the 1% and 5% tails of the minimum rank distribution. Symbols *, ** and *** denote support from >95%, >97.5% and >99% of bootstrap replicate, respectively.
(0.03 MB XLS)

Table S4 Numbers of SNPs in each category (genic, NS, non-genic) and in each population set.
(0.02 MB XLS)

Text S1 Descriptive information about populations included in this study.
(0.76 MB DOC)

Text S2 Manhattan plots showing the log_10_ BFs for each variable and for each population set.
(0.05 MB DOCX)

Text S3 Descriptive information about population subsets and comparison to worldwide sample.
(2.20 MB DOC)

## References

[1] Allen JA (1877). The influence of Physical conditions in the genesis of species.. Radical Review.

[2] Bergmann C (1847). Über die Verhältnisse der wärmeökonomie der Thiere zu ihrer Grösse.. Göttinger Studien.

[3] Gloger CL (1833). Das Abändern der Vögel durch Einfluss des Klimas..

[4] Allee W, Park C, Emerson A, Park T, Schmidt K (1949). Principles of animal ecology..

[5] Brown JH, Lee AK (1969). Bergmann's rule and climatic adaptation in woodrats (*Neotoma*).. Evolution.

[6] Johnston RE, Selander RK (1971). Evolution in the house sparrow II. Adaptive differentiation in North American populations.. Evolution.

[7] Storz JF, Balasingh J, Bhat HR, Nathan PT, Doss DPS (2001). Clinal variation in body size and sexual dimorphism in an Indian fruit bat, Cynopterus sphinx (Chiroptera: Pteropodidae).. Biological Journal of the Linnean Society.

[8] Ashton KG, Tracy MC, de Queiroz A (2000). Is Bergmann's rule valid for mammals?. American Naturalist.

[9] Freckleton RP, Harvey PH, Pagel M (2003). Bergmann's rule and body size in mammals.. American Naturalist.

[10] Harcourt AH, Schreier BM (2009). Diversity, Body Mass, and Latitudinal Gradients in Primates.. International Journal of Primatology.

[11] Mayr E (1963). Animal species and evolution..

[12] Huxley J (1938). Clines: an Auxiliary Taxonomic Principle.. Nature.

[13] Katzmarzyk PT, Leonard WR (1998). Climatic influences on human body size and proportions: ecological adaptations and secular trends.. Am J Phys Anthropol.

[14] Roberts DF (1953). Body weight, race and climate.. Am J Phys Anthropol.

[15] Leonard WR, Sorensen MV, Galloway VA, Spencer GJ, Mosher MJ (2002). Climatic influences on basal metabolic rates among circumpolar populations.. Am J Hum Biol.

[16] Jablonski NG, Chaplin G (2000). The evolution of human skin coloration.. J Hum Evol.

[17] Relethford JH (2002). Apportionment of global human genetic diversity based on craniometrics and skin color.. Am J Phys Anthropol.

[18] Loomis WF (1967). Skin-pigment regulation of vitamin-D biosynthesis in man.. Science.

[19] Chaplin G, Jablonski NG (2009). Vitamin D and the evolution of human depigmentation.. Am J Phys Anthropol.

[20] Beckman G, Birgander R, Sjalander A, Saha N, Holmberg PA (1994). Is p53 polymorphism maintained by natural selection?. Hum Hered.

[21] Cavalli-Sforza LL, Menozzi P, Piazza A (1994). History and geography of human genes..

[22] Thompson EE, Kuttab-Boulos H, Witonsky D, Yang L, Roe BA (2004). CYP3A variation and the evolution of salt-sensitivity variants.. Am J Hum Genet.

[23] Hancock AM, Clark VJ, Qian Y, Rienzo AD (2011). Population Genetic Analysis of the Uncoupling Proteins Supports a Role for UCP3 in Human Cold Resistance.. Mol Biol Evol.

[24] Young JH, Chang YP, Kim JD, Chretien JP, Klag MJ (2005). Differential susceptibility to hypertension is due to selection during the out-of-Africa expansion.. PLoS Genet.

[25] Hancock AM, Witonsky DB, Gordon AS, Eshel G, Pritchard JK (2008). Adaptations to climate in candidate genes for common metabolic disorders.. PLoS Genet.

[26] Costa R, Peixoto AA, Barbujani G, Kyriacou CP (1992). A latitudinal cline in a Drosophila clock gene.. Proc Biol Sci.

[27] Oakeshott JG, Wilson SR, Knibb WR (1988). Selection affecting enzyme polymorphisms in enclosed Drosophila populations maintained in a natural environment.. Proc Natl Acad Sci U S A.

[28] Schmidt PS, Duvernell DD, Eanes WF (2000). Adaptive evolution of a candidate gene for aging in Drosophila.. Proc Natl Acad Sci U S A.

[29] Sezgin E, Duvernell DD, Matzkin LM, Duan Y, Zhu CT (2004). Single-locus latitudinal clines and their relationship to temperate adaptation in metabolic genes and derived alleles in Drosophila melanogaster.. Genetics.

[30] Verrelli BC, Eanes WF (2001). Clinal variation for amino acid polymorphisms at the Pgm locus in Drosophila melanogaster.. Genetics.

[31] Balasubramanian S, Sureshkumar S, Agrawal M, Michael TP, Wessinger C (2006). The PHYTOCHROME C photoreceptor gene mediates natural variation in flowering and growth responses of Arabidopsis thaliana.. Nat Genet.

[32] Caicedo AL, Stinchcombe JR, Olsen KM, Schmitt J, Purugganan MD (2004). Epistatic interaction between Arabidopsis FRI and FLC flowering time genes generates a latitudinal cline in a life history trait.. Proc Natl Acad Sci U S A.

[33] Stinchcombe JR, Weinig C, Ungerer M, Olsen KM, Mays C (2004). A latitudinal cline in flowering time in Arabidopsis thaliana modulated by the flowering time gene FRIGIDA.. Proc Natl Acad Sci U S A.

[34] Grivet D, Sebastiani F, Alia R, Bataillon T, Torre S (2010). Molecular footprints of local adaptation in two Mediterranean conifers.. Mol Biol Evol.

[35] Gonzalez J, Karasov TL, Messer PW, Petrov DA (2010). Genome-wide patterns of adaptation to temperate environments associated with transposable elements in Drosophila.. PLoS Genet.

[36] Eckert AJ, van Heervaarden J, Wegrzyn JL, Nelson CD, Ross-Ibarra J (2010). Patterns of Population Structure and Environmental Associations to Aridity Across the Range of Loblolly Pine (Pinus taeda L., Pinaceae).. Genetics.

[37] Luca F, Bubba G, Basile M, Brdicka R, Michalodimitrakis E (2008). Multiple advantageous amino acid variants in the NAT2 gene in human populations.. PLoS ONE.

[38] Perry GH, Dominy NJ, Claw KG, Lee AS, Fiegler H (2007). Diet and the evolution of human amylase gene copy number variation.. Nat Genet.

[39] Hancock AM, Witonsky DB, Ehler E, Alkorta-Aranburu G, Beall C (2010). Human adaptations to diet, subsistence, and ecoregion are due to subtle shifts in allele frequency.. Proc Natl Acad Sci U S A.

[40] Endler JA (1977). Geographic variation, speciation, and clines..

[41] Akey JM, Zhang G, Zhang K, Jin L, Shriver MD (2002). Interrogating a high-density SNP map for signatures of natural selection.. Genome Res.

[42] Barreiro LB, Laval G, Quach H, Patin E, Quintana-Murci L (2008). Natural selection has driven population differentiation in modern humans.. Nat Genet.

[43] Coop G, Pickrell JK, Novembre J, Kudaravalli S, Li J (2009). The role of geography in human adaptation.. PLoS Genet.

[44] Pickrell JK, Coop G, Novembre J, Kudaravalli S, Li JZ (2009). Signals of recent positive selection in a worldwide sample of human populations.. Genome Res.

[45] Sabeti PC, Schaffner SF, Fry B, Lohmueller J, Varilly P (2006). Positive natural selection in the human lineage.. Science.

[46] Tang K, Thornton KR, Stoneking M (2007). A new approach for using genome scans to detect recent positive selection in the human genome.. PLoS Biol.

[47] Voight BF, Kudaravalli S, Wen X, Pritchard JK (2006). A map of recent positive selection in the human genome.. PLoS Biol.

[48] Fumagalli M, Pozzoli U, Cagliani R, Comi GP, Bresolin N (2010). Genome-wide identification of susceptibility alleles for viral infections through a population genetics approach.. PLoS Genet.

[49] Li JZ, Absher DM, Tang H, Southwick AM, Casto AM (2008). Worldwide human relationships inferred from genome-wide patterns of variation.. Science.

[50] Coop G, Witonsky D, Di Rienzo A, Pritchard JK (2010). Using Environmental Correlations to Identify Loci Underlying Local Adaptation.. Genetics.

[51] Jakobsson M, Scholz SW, Scheet P, Gibbs JR, VanLiere JM (2008). Genotype, haplotype and copy-number variation in worldwide human populations.. Nature.

[52] Rosenberg NA, Pritchard JK, Weber JL, Cann HM, Kidd KK (2002). Genetic structure of human populations.. Science.

[53] Albrechtsen A, Nielsen FC, Nielsen R (2010). Ascertainment biases in SNP chips affect measures of population divergence.. Mol Biol Evol.

[54] Woo R, Daniels-Kush R, Horton ES (1985). Regulation of energy balance.. Annu Rev Nutr.

[55] Worley MJ, Nieman GS, Geddes K, Heffron F (2006). Salmonella typhimurium disseminates within its host by manipulating the motility of infected cells.. Proc Natl Acad Sci U S A.

[56] Chastre E, Abdessamad M, Kruglov A, Bruyneel E, Bracke M (2009). TRIP6, a novel molecular partner of the MAGI-1 scaffolding molecule, promotes invasiveness.. FASEB J.

[57] Berry A, Kreitman M (1993). Molecular analysis of an allozyme cline: alcohol dehydrogenase in Drosophila melanogaster on the east coast of North America.. Genetics.

[58] Lao O, de Gruijter JM, van Duijn K, Navarro A, Kayser M (2007). Signatures of positive selection in genes associated with human skin pigmentation as revealed from analyses of single nucleotide polymorphisms.. Ann Hum Genet.

[59] Norton HL, Kittles RA, Parra E, McKeigue P, Mao X (2007). Genetic evidence for the convergent evolution of light skin in Europeans and East Asians.. Mol Biol Evol.

[60] Tishkoff SA, Reed FA, Ranciaro A, Voight BF, Babbitt CC (2007). Convergent adaptation of human lactase persistence in Africa and Europe.. Nat Genet.

[61] Jeon YJ, Kim DH, Jung H, Chung SJ, Chi SW (2010). Annexin A4 interacts with the NF-kappaB p50 subunit and modulates NF-kappaB transcriptional activity in a Ca2+-dependent manner.. Cell Mol Life Sci.

[62] Miao Y, Cai B, Liu L, Yang Y, Wan X (2009). Annexin IV is differentially expressed in clear cell carcinoma of the ovary.. Int J Gynecol Cancer.

[63] Kim A, Enomoto T, Serada S, Ueda Y, Takahashi T (2009). Enhanced expression of Annexin A4 in clear cell carcinoma of the ovary and its association with chemoresistance to carboplatin.. Int J Cancer.

[64] Zimmermann U, Balabanov S, Giebel J, Teller S, Junker H (2004). Increased expression and altered location of annexin IV in renal clear cell carcinoma: a possible role in tumour dissemination.. Cancer Lett.

[65] Barreiro LB, Quintana-Murci L (2010). From evolutionary genetics to human immunology: how selection shapes host defence genes.. Nat Rev Genet.

[66] Roberts DF (1978). Climate and Human Variability. 2nd edition..

[67] Kamangar F, Dores GM, Anderson WF (2006). Patterns of cancer incidence, mortality, and prevalence across five continents: defining priorities to reduce cancer disparities in different geographic regions of the world.. J Clin Oncol.

[68] Karin M, Greten FR (2005). NF-kappaB: linking inflammation and immunity to cancer development and progression.. Nat Rev Immunol.

[69] Guernier V, Hochberg ME, Guegan JF (2004). Ecology drives the worldwide distribution of human diseases.. PLoS Biol.

[70] Hancock AM, Alkorta-Aranburu G, Witonsky DB, Di Rienzo A (2010). Adaptations to new environments in humans: the role of subtle allele frequency shifts.. Philos Trans R Soc Lond B Biol Sci.

[71] Pritchard JK, Pickrell JK, Coop G (2009). The genetics of human adaptation: hard sweeps, soft sweeps, and polygenic adaptation..

[72] Kudaravalli S, Veyrieras JB, Stranger BE, Dermitzakis ET, Pritchard JK (2009). Gene expression levels are a target of recent natural selection in the human genome.. Mol Biol Evol.

[73] Branda RF, Eaton JW (1978). Skin color and nutrient photolysis: an evolutionary hypothesis.. Science.

[74] Jablonski NG (2004). The evolution of human skin and skin color.. Annual Review of Anthropology.

[75] Kovats RS, Hajat S (2008). Heat stress and public health: a critical review.. Annu Rev Public Health.

[76] Wells JC, Cole TJ (2002). Birth weight and environmental heat load: a between-population analysis.. Am J Phys Anthropol.

[77] Leonard WR, Snodgrass JJ, Sorensen MV (2005). Metabolic adaptation in indigenous Siberian populations.. Annual Review of Anthropology.

[78] Beall CM, Steegman AT, Stinson S, Bogin B, Huss-Ashmore R, O'Rourke D (2000). Human Adaptation to Climate: Temperature, Ultraviolet Radiation and Altitude.. Human Biology: An Evolutionary and Biocultural Perspective.

[79] Bustamante CD, Fledel-Alon A, Williamson S, Nielsen R, Hubisz MT (2005). Natural selection on protein-coding genes in the human genome.. Nature.

[80] Przeworski M, Hudson RR, Di Rienzo A (2000). Adjusting the focus on human variation.. Trends Genet.

[81] Di Rienzo A (2006). Population genetics models of common diseases.. Curr Opin Genet Dev.

[82] Williams GC (1957). Pleiotropy, natural-selection, and the evolution of senescence.. Evolution.

[83] Barreiro LB, Ben-Ali M, Quach H, Laval G, Patin E (2009). Evolutionary dynamics of human Toll-like receptors and their different contributions to host defense.. PLoS Genet.

[84] Karin M, Cao Y, Greten FR, Li ZW (2002). NF-kappaB in cancer: from innocent bystander to major culprit.. Nat Rev Cancer.

[85] Kistler R, Kalnay E, Collins W, Saha S, White G (2001). The NCEP-NCAR 50-year reanalysis: Monthly means CD-ROM and documentation.. Bulletin of the American Meteorological Society.

[86] Sellke T, Bayarri MJ, Berger JO (2001). Calibration of p values for testing precise null hypotheses.. American Statistician.

[87] Stephens M, Balding DJ (2009). Bayesian statistical methods for genetic association studies.. Nat Rev Genet.

[88] Eberle MA, Ng PC, Kuhn K, Zhou L, Peiffer DA (2007). Power to detect risk alleles using genome-wide tag SNP panels.. PLoS Genet.

[89] Hindorff LA, Junkins HA, Mehta JP, Manolio TA (2009). A Catalog of Published Genome-Wide Association Studies.. http://www.genome.gov/gwastudies.

[90] Becker KG, Barnes KC, Bright TJ, Wang SA (2004). The genetic association database.. Nat Genet.

[91] Fielding AH (2007). Cluster and Classification Techniques for the Biosciences..

