# Supplementary figures and images for: Adaptations to Climate-Mediated Selective Pressures in Humans
Source: PLoS Genet. 2011 Apr 21;7(4):e1001375. doi: 10.1371/journal.pgen.1001375 (PMC3080864; doi:10.1371/journal.pgen.1001375)

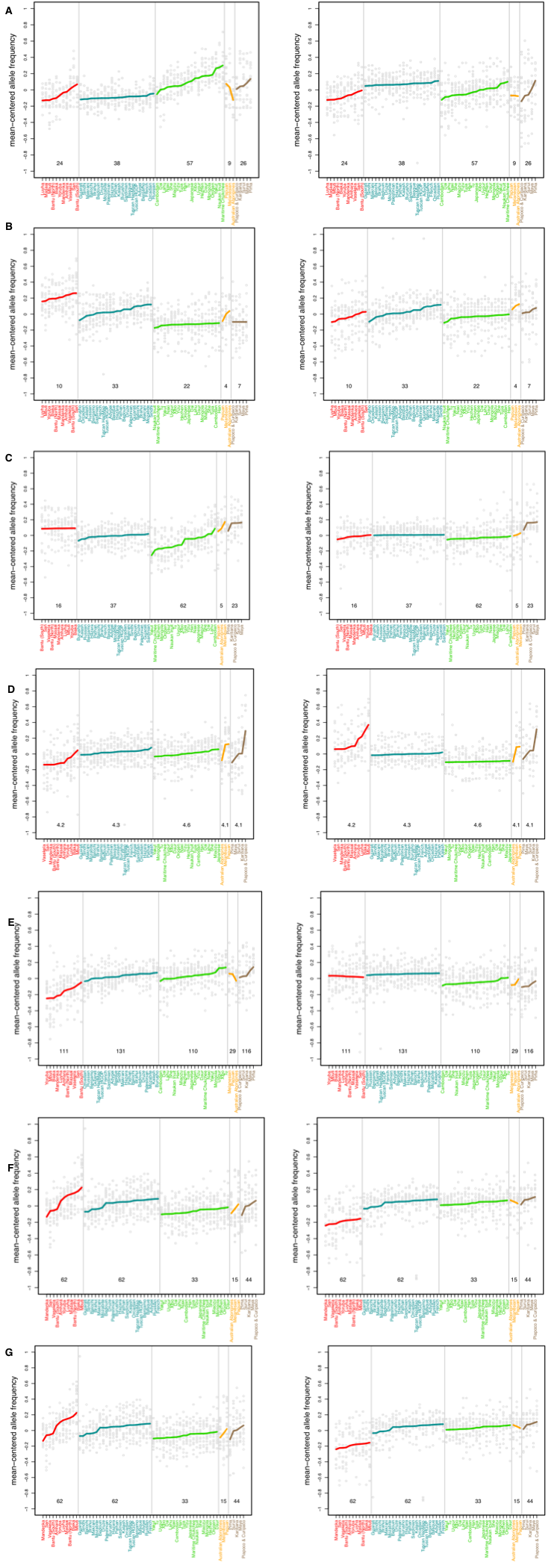

Supplement: Figure S1 — Transformed allele frequency plotted against each of seven climate variables for SNPs with the strongest signals in the worldwide analysis. Since the particular patterns that result in strong correlations in the worldwide analysis are diverse, SNPs for these variables were split into two clusters using the results of an eigen analysis of the matrix of SNPs and populations. SNPs were assigned to clusters based on the eigenvector term for the eigenvector corresponding to the first eigenvalue. Panels include: (A) absolute latitude, (B) maximum summer temperature, (C) minimum winter temperature, (D) winter precipitation rate, (E) summer solar radiation, (F) summer relative humidity, and (G) winter relative humidity. Transformed allele frequencies were computed by subtracting the mean allele frequency across populations. SNPs with rank statistics less than 1e-5 are included in the plots. Population names and means are colored based on membership in one of seven major geographical regions (sub-Saharan Africa, Europe, Middle East, West Asia, East Asia, Oceania, or the Americas) and ordered so that the climate variable values increase from left to right across the x-axis. Each gray dot represents an individual SNP and fitted lines for each region are shown in color. The range of each climate variable across the geographic region is shown in each section of the plot. (6.30 MB TIF) [file pgen.1001375.s001.tif]

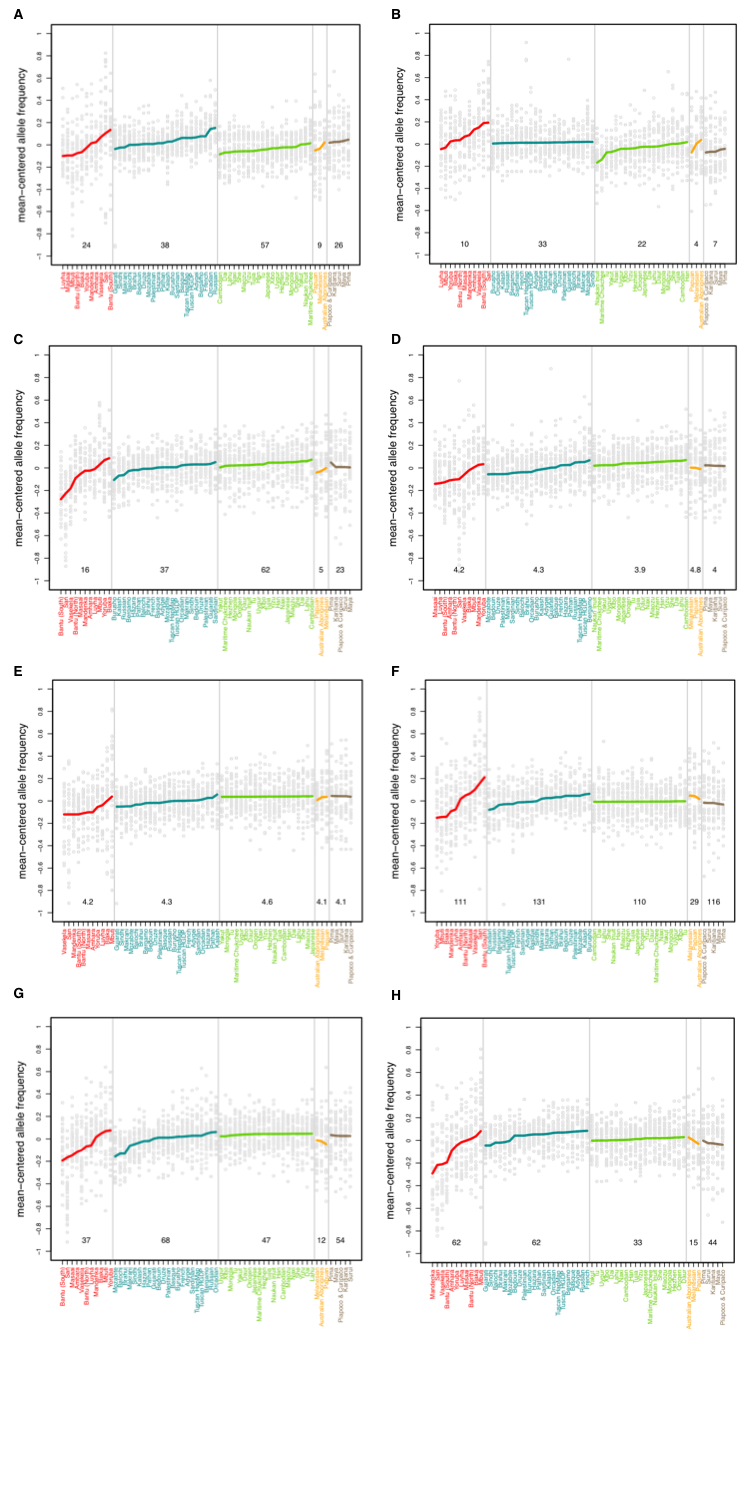

Supplement: Figure S2 — Transformed allele frequency plotted against each of eight climate variables for SNPs with the strongest signatures of selection in the AWE population subset. Panels include: (A) absolute latitude, (B) maximum summer temperature, (C) minimum winter temperature, (D) summer precipitation rate, (E) winter precipitation rate, (F) summer solar radiation, (G) summer relative humidity, and (H) winter relative humidity. Transformed allele frequencies were computed by subtracting the mean allele frequency across populations. SNPs with rank statistics less than 1e-5 are included in the plots. Population names and means are colored based on membership in one of seven major geographical regions (sub-Saharan Africa, Europe, Middle East, West Asia, East Asia, Oceania, or the Americas) and ordered so that the climate variable values increase from left to right across the x-axis. Each gray dot represents an individual SNP and fitted lines for each region are shown in color. The range of each climate variable across the geographic region is shown in each section of the plot. (4.50 MB TIF) [file pgen.1001375.s002.tif]

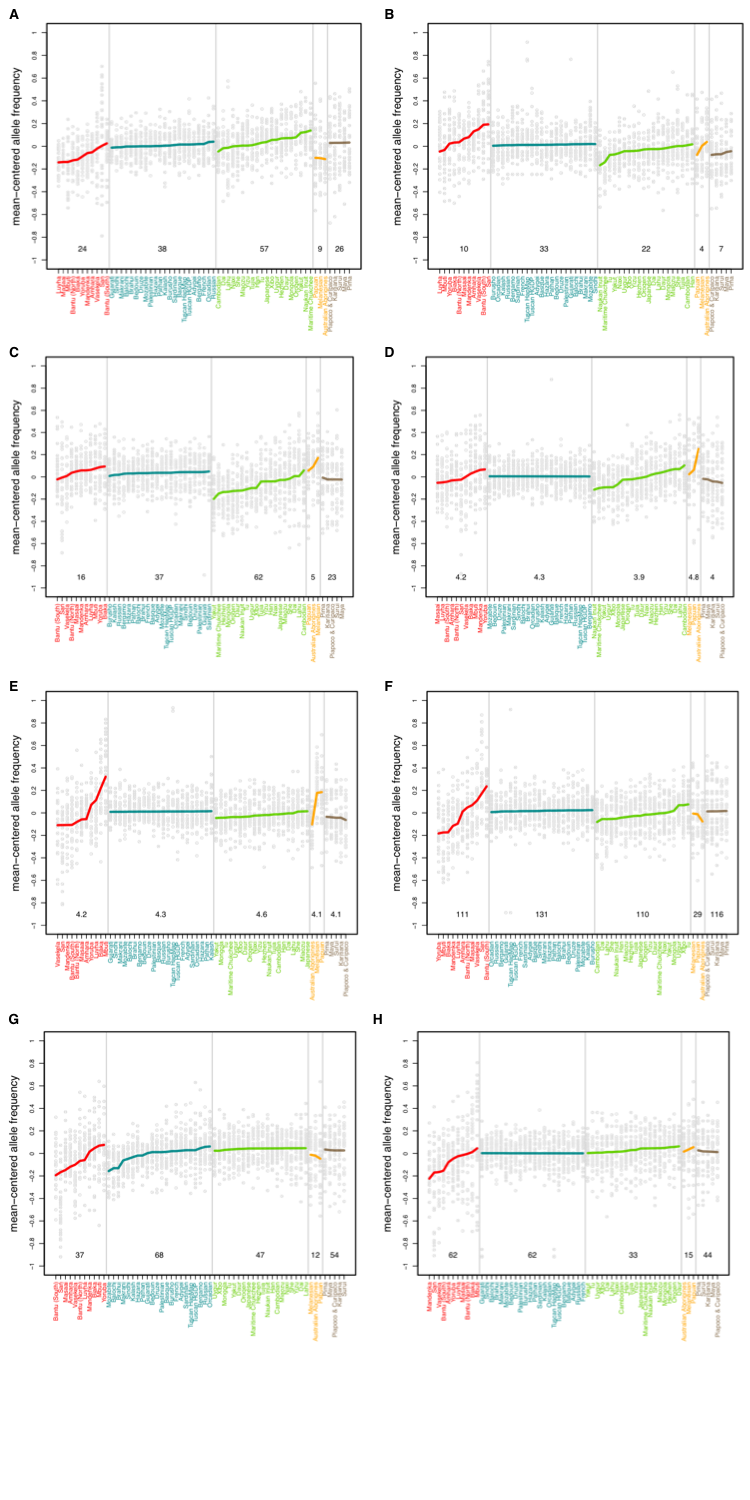

Supplement: Figure S3 — Transformed allele frequency plotted against each of eight climate variables for SNPs with the strongest signatures of selection in the AEA population subset. Panels include: (A) absolute latitude, (B) summer maximum temperature, (C) winter minimum temperature, (D) summer precipitation rate, (E) winter precipitation rate, (F) summer solar radiation, (G) summer relative humidity, and (H) winter relative humidity. Transformed allele frequencies were computed by subtracting the mean allele frequency across populations. SNPs with rank statistics less than 1e-5 are included in the plots. Population names and means are colored based on membership in one of seven major geographical regions (sub-Saharan Africa, Europe, Middle East, West Asia, East Asia, Oceania, or the Americas) and ordered so that the climate variable values increase from left to right across the x-axis. Each gray dot represents an individual SNP and fitted lines for each region are shown in color. The range of each climate variable across the geographic region is shown in each section of the plot. (4.50 MB TIF) [file pgen.1001375.s003.tif]

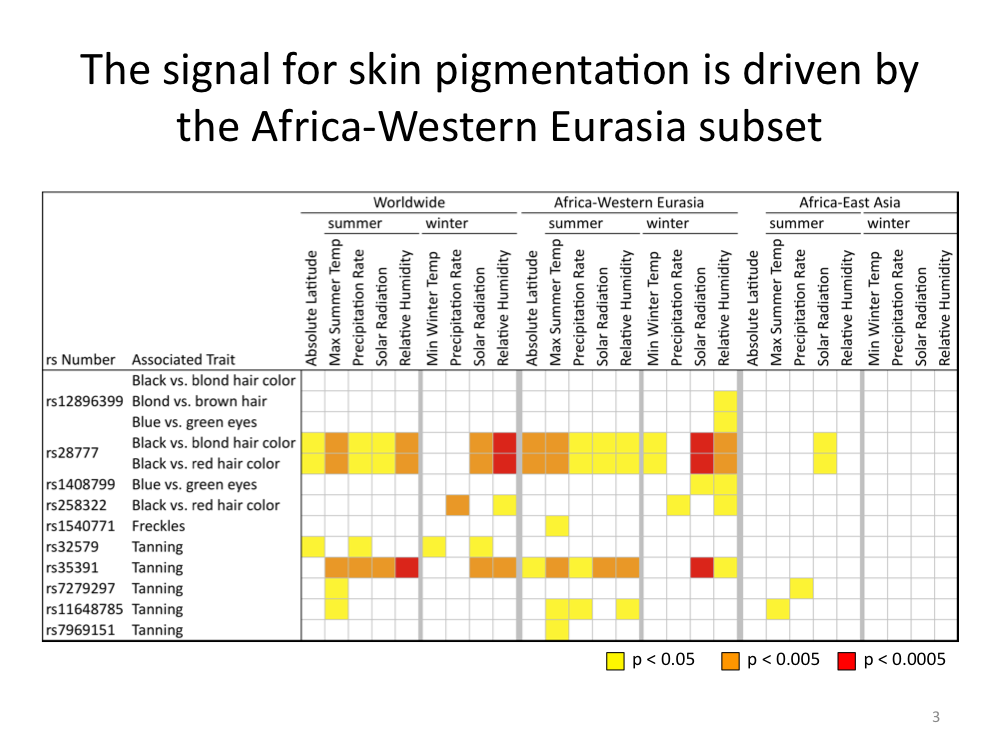

Supplement: Figure S4 — Two SNPs implicated in pigmentation phenotypes that have strong correlations with winter solar radiation in the AWE population subset. (A) rs1667394, a SNP in OCA2, and (B) rs28777, a SNP in SLC45A2. (3.00 MB TIF) [file pgen.1001375.s004.tif]
