# Supplementary material for: Adaptations to Climate-Mediated Selective Pressures in Humans
Source: PLoS Genet. 2011 Apr 21;7(4):e1001375. doi: 10.1371/journal.pgen.1001375 (PMC3080864; doi:10.1371/journal.pgen.1001375)

Information about populations included in the analysis that are not part of the Human Genome Diversity Project panel

| Population | Source | Geographic origin | Coordinates | Number individuals genotypes | Number individuals after relatives removed |
| --- | --- | --- | --- | --- | --- |
| Luhya | HapMap Phase 3 | Webuye, Kenya | 0.5N, 35E | 83 | 71 |
| Maasai | HapMap Phase 3 | Kinyawa, Kenya | 1S, 36E | 141 | 61 |
| Toscani | HapMap Phase 3 | Tuscany, Italy | 43N, 11E | 77 | 77 |
| Gujarati | HapMap Phase 3 | Houston, Texas, but originally from Gujarat Province, India | 23N, 71E | 83 | 79 |
| Amhara | Cynthia Beall, Case Western Reserve University; Amha Gebremedhin, Addis Ababa University, Ethiopia | Ethiopia | 13N, 38E | 23 | 22 |
| Vasakela | Gerd Utermann, Innsbruck Medical University | Collected in Schmidtsdrift, South Africa, but individuals are originally from Angola | 17.5S, 22E | 24 | 22 |
| Maritime Chukchi | Rem Sukernik, Russian Academy of Sciences | Siberia | 69N, 170E | 25 | 22 |
| Naukan Yu’pik | Rem Sukernik, Russian Academy of Sciences | Siberia | 65N, 172W | 23 | 22 |
| Australian Aborigines | European Collection of Cell Cultures (ECACC) Ethnic Diversity DNA Panel | Australia | 13S, 143E | 10 | 8 |

Neighbor-joining tree of populations


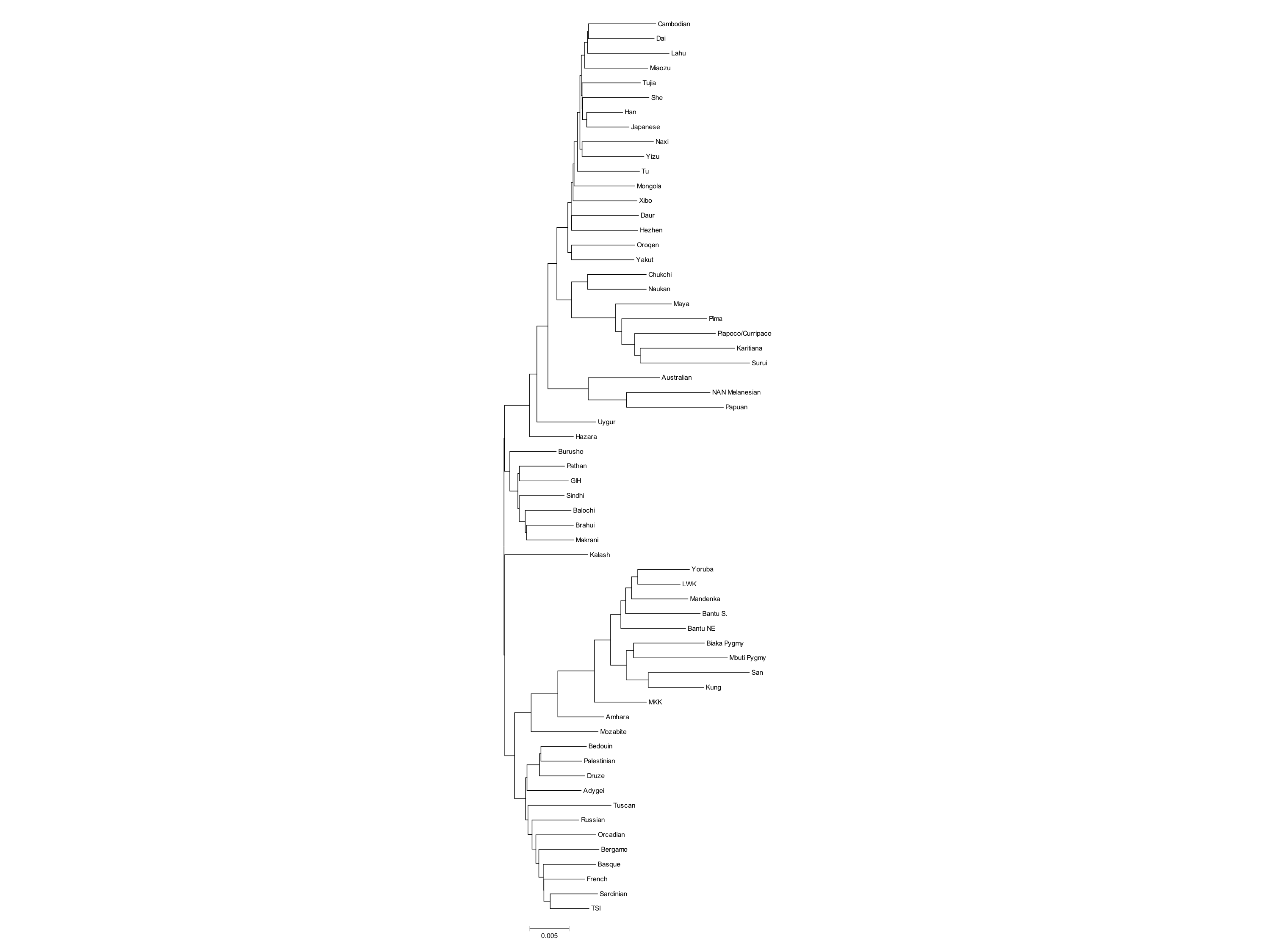


Correlation matrices based on covariance matrices of populations
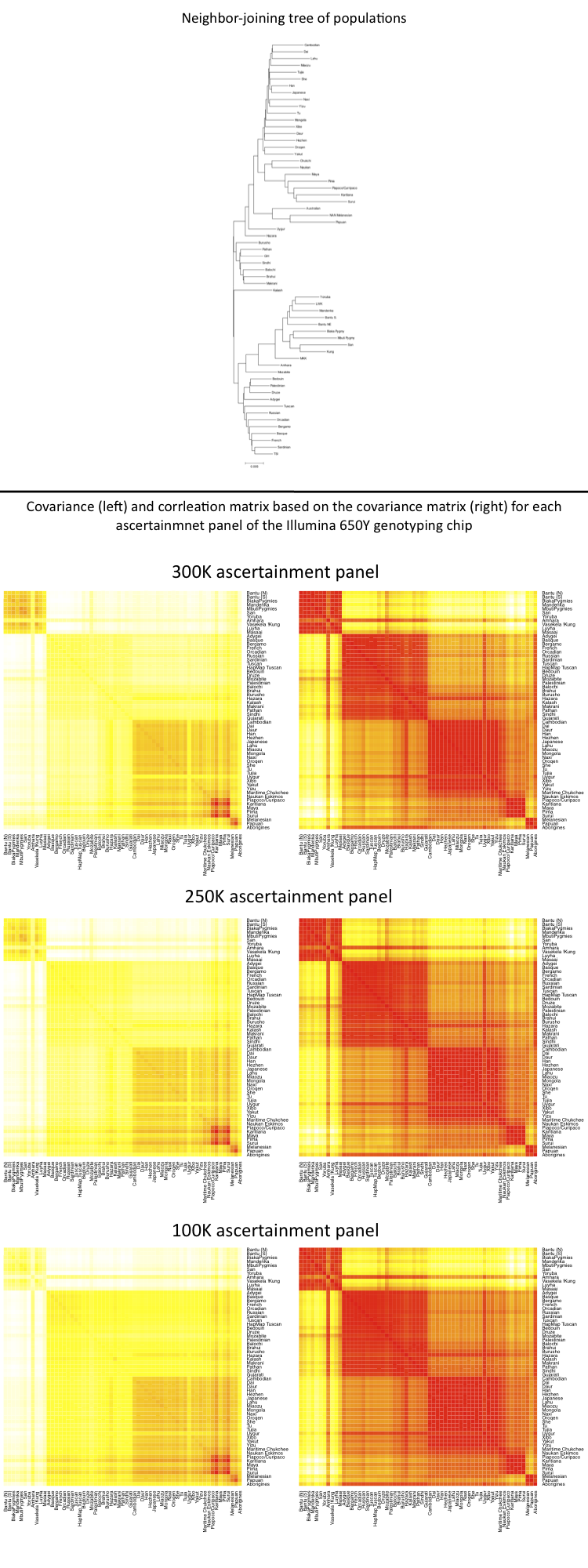

Supplement: Text S1 — Descriptive information about populations included in this study. (0.76 MB DOC) [file pgen.1001375.s012.doc]
