# Supplementary material for: Adaptations to Climate-Mediated Selective Pressures in Humans
Source: PLoS Genet. 2011 Apr 21;7(4):e1001375. doi: 10.1371/journal.pgen.1001375 (PMC3080864; doi:10.1371/journal.pgen.1001375)
Supplement: Text S2 — Manhattan plots showing the log10 BFs for each variable and for each population set. (0.05 MB DOCX) [file pgen.1001375.s013.docx]

Membership in population subsets

| Supplementary Table 2. Membership in population subsets | | |
| --- | --- | --- |
|  |  | |
| Population | AWE | AEA |
| Bantu (North) | X | X |
| Bantu (South) | X | X |
| Biaka Pygmies | X | X |
| Mandenka | X | X |
| Mbuti Pygmies | X | X |
| San | X | X |
| Yoruba | X | X |
| Amhara | X | X |
| Vasakela | X | X |
| Luyha | X | X |
| Masaai | X | X |
| Adygei | X |  |
| Basque | X |  |
| Bergamo | X |  |
| French | X |  |
| Orcadian | X |  |
| Russian | X |  |
| Sardinian | X |  |
| Tuscan | X |  |
| HapMap Tuscans | X |  |
| Bedouin | X | X |
| Druze | X | X |
| Mozabite | X | X |
| Palestinian | X | X |
| Balochi | X |  |
| Brahui | X |  |
| Burusho | X |  |
| Hazara |  |  |
| Kalash | X |  |
| Makrani | X |  |
| Pathan | X |  |
| Sindhi | X |  |
| Uygur |  |  |
| Xibo | X |  |
| Gujarati | X |  |
| Cambodian |  | X |
| Dai |  | X |
| Daur |  | X |
| Han |  | X |
| Hezhen |  | X |
| Japanese |  | X |
| Lahu |  | X |
| Miaozu |  | X |
| Mongola |  | X |
| Naxi |  | X |
| Oroqen |  | X |
| She |  | X |
| Tu |  | X |
| Tujia |  | X |
| Yakut |  | X |
| Yizu |  | X |
| Maritime Chukchee |  | X |
| Naukan Yup’ik |  | X |
| Colombian (Piapoco/Curipaco) |  |  |
| Karitiana |  |  |
| Maya |  |  |
| Pima |  |  |
| Surui |  |  |
| Melanesian |  | X |
| Papuan |  | X |
| Aborigines |  | X |

Overlap between SNPs in the tails for population subset and worldwide climate analysis.

| Tail Cutoff | Overlap between Worldwide Sample and: | | Overlap expected by chance^a^ |
| --- | --- | --- | --- |
|  | AWE | AEA |  |
| 5% | 6778 | 5770 | 1320 |
| 1% | 796 | 823 | 53 |
| 0.50% | 330 | 353 | 13 |
| 0.10% | 38 | 41 | 0.53 |
| 0.05% | 16 | 22 | 0.13 |
