# Supplementary material for: Adaptations to Climate-Mediated Selective Pressures in Humans
Source: PLoS Genet. 2011 Apr 21;7(4):e1001375. doi: 10.1371/journal.pgen.1001375 (PMC3080864; doi:10.1371/journal.pgen.1001375)

Manhattan plots showing the log_10_BFs for each variable in the worldwide analysis.


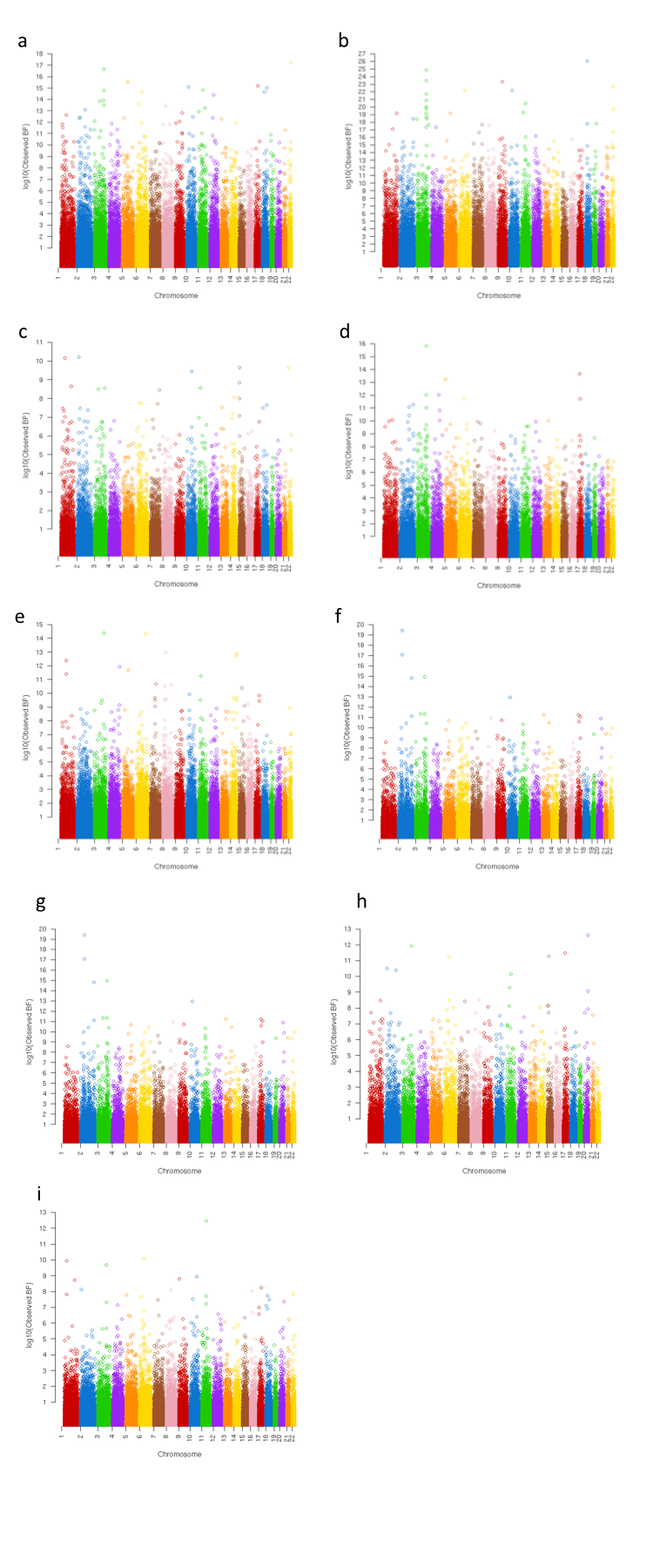


Manhattan plots showing the log_10_BFs for each variable in the AWE population subset analysis.


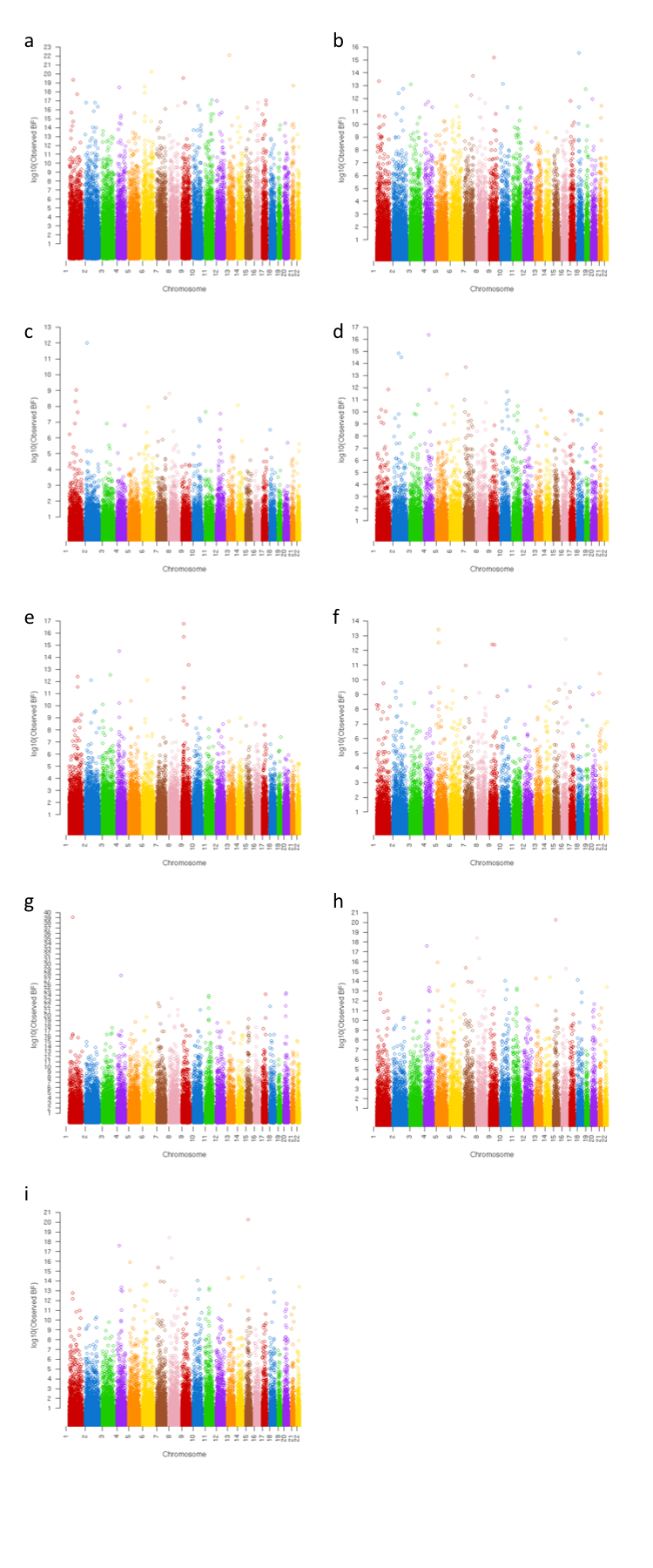


Manhattan plots showing the log_10_BFs for each variable in the AEA population subset analysis.


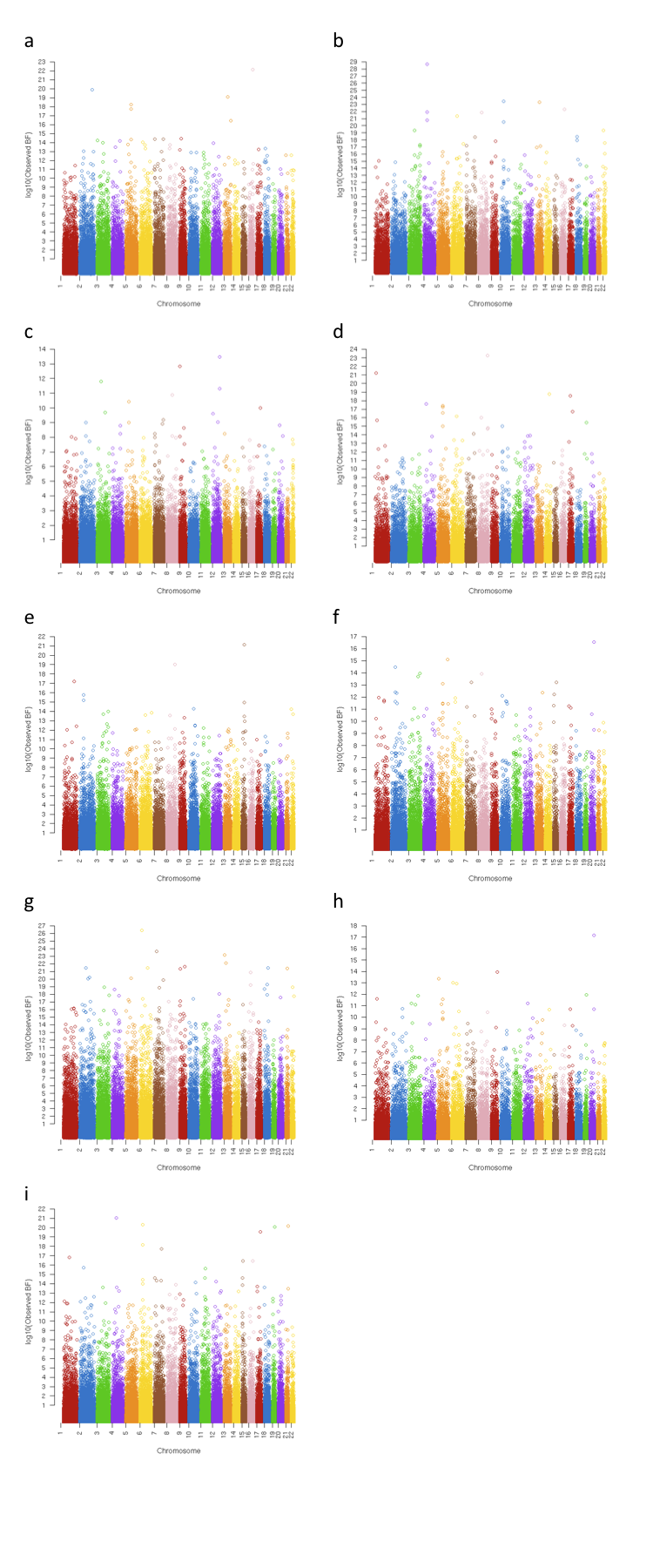

Supplement: Text S3 — Descriptive information about population subsets and comparison to worldwide sample. (2.20 MB DOC) [file pgen.1001375.s014.doc]
